# Supplementary material for: In-ear integrated sensor array for the continuous monitoring of brain activity and of lactate in sweat
Source: Nat Biomed Eng. 2023 Sep 28;7(10):1307–20. doi: 10.1038/s41551-023-01095-1 (PMC10589098; doi:10.1038/s41551-023-01095-1)
Supplement: Supplementary file 1 — Supplementary methods, discussion, figures, tables, references and video captions. [file 41551_2023_1095_MOESM1_ESM.pdf]

nature biomedical engineering

Article https://doi.org/10.1038/s41551-023-01095-1

In-ear integrated sensor array for the continuous monitoring of brain activity and of lactate in sweat

In the format provided by the authors and unedited

nature biomedical engineering

Article https://doi.org/10.1038/s41551-023-01095-1

In-ear integrated sensor array for the continuous monitoring of brain activity and of lactate in sweat

In the format provided by the authors and unedited

List of abbreviations 2
Supplementary Note 1 | Design and fabrication of the sensors 3
Preparations of the materials 3
Printing procedures 3
Three-dimensional electrophysiological electrode simulation and fabrication 3
Bonding procedures 3
Electrochemical lactate electrode modification and working mechanism 4
Polyvinyl alcohol-hydrogel preparation procedures 4
Sensors' chemical component analysis 4
Supplementary Note 2 | Characterization of the robustness of the sensors 5
Mechanical robustness 5
Robustness under different temperature and humidity conditions 5
Supplementary Note 3 | Characterization of electrophysiological sensing 7
Electrode-ear impedance equivalent circuit models 7
Eye movement signal measurement results and analysis 7
Electrophysiological sensing validation 8
Simultaneous electrophysiological sensing 9
EEG Motion artifact reduction 10
Supplementary Note 4 | Characterization of the combined electrophysiological and electrochemical sensing crosstalk 12
EEG sensing to lactate sensing crosstalk analysis 12
Lactate sensing to EEG sensing crosstalk analysis 12
Spectral analyses of the EEG with chronoamperometric induced artifacts 13
Supplementary Note 5 | Evaluation of usability and wearability 15
Supplementary Figures 16
Supplementary Tables 31
Supplementary Videos 35
Supplementary Video 1 | Eye movement sensing demonstration 35
Supplementary Video 2 | Lactate sensing demonstration 35
Supplementary Video 3 | Mechanical stability demonstration 35
Supplementary references 36

List of abbreviations

AA	Ascorbic acid
AC	Acetaminophen
ADC	Analog to digital converter
Ag	Silver
ASSR	Auditory steady-state response
ASR	Automatic subspace reconstruction
AVDD	Analog positive supply voltage (MOSFET drain power supply voltage)
AVSS	Analog negative supply voltage (MOSFET source power supply voltage)
BSA	Bovine serum albumin
CA	Chronoamperometry
CE	Counter electrode
CPE	Constant phase element
DAQ	Data acquisition
DI	Deionized
DRL	Driven right leg
ECG	Electrocardiography
eChem	Electrochemical
EDA	Electrodermal activity
EDO	Electrode DC offset
EDS	Energy-dispersive X-ray spectroscopy
EEG	Electroencephalography
EIS	Electrochemical impedance spectroscopy
EMG	Electromyography
EOG	Electrooculography
ePhys	Electrophysiological
FBCSP	Filter-bank-based common-spatial-pattern
fPCB	Flexible printed circuit board
FTIR	Fourier-transform infrared
Gluc	Glucose
ICA	Independent component analysis
KOH	Potassium hydroxide
LA	Lactic acid
LOx	Lactate oxidase
PB	Prussian blue
PBS	Phosphate-buffered saline
PCB	Printed circuit board
PGA	Programmable gain amplifier
PLA	Polylactic acid
PSD	Power spectral density
PVA	Polyvinyl alcohol
RE	Reference electrode (for electrochemical sensors and EIS)
REF	Reference electrode (for electrophysiological sensors)
SEBS	Styrene-ethylene-butylene-styrene block copolymer
SEM	Scanning electron microscope
SNR	Signal-to-noise ratio
SVM	Support-vector-machine
TPU	Terephthalate polyurethane
UA	Uric acid
WE	Working electrode

Supplementary Note 1 I Design and fabrication of the sensors

Preparations of the materials

Styrene-ethylene-butylene-styrene block copolymer (SEBS): The SEBS resin was prepared by dispersing SEBS beads into toluene (weight ratio of 1: 2). The mixture was mixed with a linear shaker (Scilogex, SK-L180-E) overnight or until the mixture became transparent and homogeneous. The cast resin was first dried in an ambient environment for 1 hr, then cured in a conventional oven at 80 °C for 1 hr to remove the excess solvent.

Stretchable Silver: The stretchable Ag ink was synthesized by mixing SEBS (4 g to 10 mL toluene ratio) and Ag flakes in a weight ratio of 1:2, in a dual asymmetric centrifugal mixer (Flacktek Speedmixer, DAC 150.1 KV-K) with a speed of 1800 RPM for 10 minutes or until obtaining a homogeneous ink.

Stretchable Prussian-blue (PB): The stretchable PB ink was synthesized by mixing super-P carbon black, graphite powder, PB, SEBS (4 g to 10 mL toluene ratio), and toluene, in a weight ratio of 0.5: 3: 1: 1.26: 4.74, in the mixer at 2150 RPM for 10 minutes or until the ink was homogeneous. Before printing the stretchable inks, the ink viscosity was analyzed visually, and if necessary, (~200 μL) toluene was added, and the ink was centrifuged before use.

Phosphate-buffered saline (PBS): PBS used in the electrode modification was prepared in 0.1 M with a pH of 7.4. All bovine serum albumin BSA solutions were prepared with a concentration of 10 mg/mL in PBS.

Chitosan: The chitosan solution was prepared by dissolving chitosan in 0.1 M acetic acid with concentration of 0.5 wt%.

Printing procedures

The fabrication of the in-ear sensors was based on a cost-effective layer-by-layer screen printing method (**Extended Data Fig. 2a–f**). The screen-printing was carried out using a semi-automatic MPM-SPM printer (Speedline Technologies). Custom stainless-steel stencils were designed (AutoCAD, Autodesk) and fabricated (Metal Etch Services) with the dimensions 10 inches × 10 inches and a thickness of 150 µm. The electrodes were printed layer-by-layer, as illustrated in **Extended Data Fig. 2m–p**. Specifically, the following printer parameters were used for each layer: stretchable Ag layer: force 40, speed 4, SEBS layer: force 20, speed 2, PB layer: force 20, speed 3. Before printing the PB layer, a layer of universal release agent (Universal mold release, Smooth-on Inc.) was sprayed onto the back of the stencil to avoid adhesion between the stencil and the SEBS. After printing, a double-sided adhesive (Medical Tape 1509, 3M Medical) was affixed to the backside of the sensor for later assembly.

Three-dimensional electrophysiological electrode simulation and fabrication

A finite element model was built to illustrate the mechanism of the 3D electrophysiological electrode formation. In the FEM, one electrophysiological electrode was modeled using the actual size of the terephthalate polyurethane (TPU) substrate and the stretchable Ag. The materials in the FEM used literature-reported parameters. For TPU: Thermal conductivity: 0.2 W/(m·K), Density: 1050 kg/m^3, Young's modulus: 0.5 GPa, Poisson's ratio: 0.42, heat capacity at constant pressure: 1800 J/(kg*K), Coefficient of thermal expansion: 1.7^{-4} 1/K[51,52]. For stretchable Ag: as an approximation, the stretchable Ag used the parameters characterized from a solution-derived nanoparticle Ag ink thin film[53,54]. Thermal conductivity: 429 W/(m·K), Density: 10500 kg/m^3, Young's modulus: 110 GPa, Poisson's ratio: 0.37, heat capacity at constant pressure: 235 J/(kg*K), Coefficient of thermal expansion: 1.91^{-4} 1/K[51]. Both materials were modeled as linear elastic materials with fixed constraints along the TPU's edges. A boundary temperature condition of 373.15 K (100°C) was applied to all the exposed surfaces of the model. The FEM sectional displacement results after applying the heat condition (**Extended Data Fig. 2s**) show a deflection of 53% of the TPU thickness (80 µm/150 µm TPU thickness).

The stretchable Ag 3D electrodes were molded using a 3D printed 750-µm-thick tough polylactic acid (PLA) mold (S5, Ultimaker) and stretchable Ag ink (**Extended Data Fig. 2t–v**). The procedures were as follows: sprayed the back of the mold with the universal release agent (Universal mold release, Smooth-on Inc.), aligned the mold over the in-ear sensors, filled each mold hole with stretchable Ag ink, scratched the excessive ink with a blazer to make the surface flat, lifted the mold as soon as possible to avoid adhesion to the mold, and cured in the oven at 100 °C for 10 mins. The following steps created the curvature structure or "holes" under each electrophysiological electrode. These "holes" were then filled with SEBS and cured in the oven at 100 °C for 10 minutes.

Bonding procedures

After manufacturing the in-ear sensors, the sensors and the 3M medical tape at its bottom were carved along its designed profile (**Extended Data Fig. 2g**). A standard polyimide substrate two-layer flexible printed circuit

board (PCB) (designed with Altium Designer, Altium Inc., Australia, and fabricated by PCBWay, China) was aligned, attached, and soldered to the silver traces using silver liquid solder aided by a Kapton tape transfer layer (**Extended Data Fig. 2h–j**). The flexible PCB has a dedicated adhesive area at the bottom side for attaching to the bonding area of the sensors (**Extended Data Fig. 2y**). After bonding the sensors and the flexible PCB, the adhesive liners of the 3M double-sided adhesive at the bottom of the sensor were released, and the sensors were attached onto the earphone's target area (**Extended Data Fig. 2k**) and secured using the 3M medical double-sided adhesive as well as a silicone earphone hook.

Electrochemical lactate electrode modification and working mechanism
For lactate electrode modification, the chitosan solution was mixed with a lactate oxidase (LOx) (40 mg/mL) in BSA solution, in a volume ratio of 1: 1, followed by drop casting a 2 μL aliquot of the mixture onto the working electrode surface modification (**Extended Data Fig. 2l**). After drying at room temperature for 1 hr, 2 μL of the chitosan solution was drop cast to the previously modified surface. Upon completing the corresponding modification steps, the resulting sensors were stored at 4°C overnight before use. The lactate sensing mechanism is based on the reaction of LOx on the working electrode surface with lactate, resulting in the creation of hydrogen peroxide and pyruvate. Further, the PB-based electrode transducer transforms the hydrogen peroxide product to hydroxyl ions (OH^-) for selective lactate detection (**Extended Data Fig. 3a,b**).

Polyvinyl alcohol-hydrogel preparation procedures
The fabrication of the porous Polyvinyl alcohol (PVA) hydrogel was based on the protocol from a previous report[55]. Solutions of PVA and potassium hydroxide (KOH) were prepared in deionized (DI) water in a weight ratio of 1:10 and 1:30, respectively. Next, 10 g of the PVA solution, 14 g of the KOH solution, and 2 mL of DI water containing 2.6 g of table sugar were mixed in a beaker under mild stirring. Afterward, 15 g of the solution was placed into a petri dish (diameter ~9 cm) and left inside a vacuum desiccator overnight to remove the excess water and allow the mixture to crosslink. The cross-linked hydrogel material (thickness: 0.5 mm) was then immersed inside a 0.1 M PBS solution to remove the sugar and excess KOH (until the gel reached a pH of 7). The gel was then cut into 1 × 1 cm^2 pieces and stored in a bottle containing 0.1 M PBS for its storage.

Sensors' chemical component analysis
Component analysis was conducted to verify the safety of the sensors to be used in the ear. For structural materials that will be in contact with the skin, the electrophysiological electrodes were made with Ag composite materials which has been widely used and considered safe. For the electrochemical sensors, PVA gel was designed to be in contact with the skin. Apart from these, SEBS was used as the insulation layer which can also touch the skin.

The PVA hydrogel materials have been assessed for the KOH contents. The KOH content of the alkaline solution from the hydrogel membranes was removed by performing repetitive washing steps with phosphate buffer (pH 7.0). During every washing cycle, the pH of the solution containing the hydrogel membranes was recorded using commercial pH strips. These washing steps were optimized by examining the KOH content using FTIR (Fourier-transform infrared) spectroscopic methods. **Supplementary Fig. 5a** shows the disappearance of the peak at ~670 cm^{-1} and confirms the absence of the K-O bond, which corresponds to the absence of the KOH from the hydrogel. In addition, the PVA hydrogel has also been assessed by using the pH measurement at its surface every time before the experimentation, where the neutral pH ensures also corroborates the removal of the KOH irritant from the PVA-hydrogel.

For the SEBS, during the patch fabrication process, the material layers are subsequently heat cured at 80°C and 60°C upon the deposition of each layer. For developing the sensor patch, a total of 30 minutes (10+10+10 minutes) of heat treatment has been given, where for the SEBS layer gets 20 minutes of heat exposure (at 80°C) in the oven, which considerably evaporates the toluene from the patch. In order to verify, we have performed the material characterization using thermogravimetric analysis (TGA) (**Supplementary Fig. 5b**), where the SEBS film has been characterized after the curing. TGA curve demonstrates the mass loss at different temperatures. The consistent 100% mass retention till the temperature of 250°C indicates the SEBS film has no toluene traces which would have otherwise been seen with mass loss at its boiling point (~110°C). The mass loss starting from ~250 °C is attributed to the heat degradation of SEBS.

Supplementary Note 2 | Characterization of the robustness of the sensors

Mechanical robustness

Since the variation of ear geometries between human participants is notable, different mechanical deformations such as bending and twisting may be applied to the sensors before, during, and after insertion. Therefore, the mechanical robustness and stability of the in-ear integrated sensors was evaluated to ensure functionality over an extended period. To improve the flexibility of the sensor, all the materials used during the screen-printing process were chosen to have stretchability. To characterize the sensors' resilience to possible insertion deformation, the sensors were tested with a repetitive dual-direction uni-axial strain of 20%. The mechanical stretching was performed in a controlled setting with a programmable motorized linear stage (X-LRQ, Zaber Technologies Inc.). The stage clamped both edges of the in-ear integrated sensors (**Fig. 1g**) and stretched with controlled strain and speed for a pre-programmed distance. One stretching cycle consisted of stretching in both the longitudinal direction and the latitudinal direction sequentially. The stretching distance was 120% the length of the direction being stretched. The stretching speed was set at 3 mm/s for both the stretching and releasing stages. Additionally, the resiliency of the sensors against insertion was visually inspected by inserting it into and retracting it out of a silicone ear phantom repetitively (**Fig. 1j**). To characterize the pre-stretching and post-stretching material structure and morphology, scanning electron microscope (SEM) images (FEI Quanta 250) were taken before and after the 100 cycles of mechanical stretching of the same in-ear integrated sensors. To characterize the electrodes' connectivity before and after stretching, pre-stretching and post-stretching impedance measurements were performed with the potentiostat in a three-electrode setup (**Supplementary Fig. 6i**). Here the working electrode (WE) was the electrophysiological or electrochemical electrode to be measured using the impedance spectroscopy method, with an E_{ac} = 10 mV and a frequency range of 1–100 kHz. The reference electrode (RE) and the counter electrode (CE) used an Ag/AgCl reference electrode (Gamry Instruments) and a platinum wire, respectively. To characterize the lactate sensing electrodes' functionality after stretching, pre-stretching and post-stretching in-vitro lactate sensing measurements were taken on each cycling by running chronoamperometry (CA) steps of 60 s under a fixed concentration of 2 mM lactate.

Supplementary Fig. 6a–h shows the pre- and post-stretching SEM microscopic structure of the electrodes on the sensors. By design, there were two types of electrodes classified by the outermost layer materials. The Ag-type electrodes included the electrophysiological electrodes and the electrochemical reference electrode, which were made with stretchable Ag. The PB-type electrodes included the electrochemical working electrode and counter electrode, which had a surface coating of PB. The SEM showed no apparent structural damage or cracks for both the Ag-type and PB-type electrodes after 100 stretching cycles.

Supplementary Fig. 6j,k shows pre- and post-stretching Ag-type and Pb-type electrode impedance measured in the 1 mol/L PBS. After stretching, the Ag-type electrode maintained a low (<2 kΩ @ 10 Hz) impedance and demonstrated a less capacitive behavior, indicated by a reduced phase angle at low frequency. The PB-type electrode retained its behavior at low frequency (<100 Hz) and demonstrated a less capacitive behavior at high frequency. In both cases, stretching the electrode structure resulted in a more porous structure, which promoted more direct charge transfers across the surface and a smaller double capacitance.

Extended Data Fig. 8b shows the pre- and post-stretching in-vitro lactate sensing performance. By comparing the chronoamperometric intensity of each stretching cycle, minimal current changes (<8%) were observed as the stretching cycles progressed. Such negligible differences demonstrate the efficient combination of stretchable components integrated into the costume inks during fabrication.

Robustness under different temperature and humidity conditions

To demonstrate the robustness and stability of the in-ear integrated sensors under varying temperature and humidity conditions inside the ear, we characterized the sensors under an expanded testing condition in comparison to the reported normal temperature (36.4 ± 0.6 °C[40]) and humidity (relative humidity: 40% - 70%[41]) profiles inside the ear. For characterization of stability, the conductivity of the electrophysiological electrodes was measured using a three-electrode electrochemical impedance spectroscopy (EIS) setup in which a droplet of 1 M PBS was placed on top of one electrophysiological electrode; the EIS setup was the same to that described in the section on mechanical robustness. The sensitivity of the electrochemical lactate electrodes was evaluated using the chronoamperometry method described in the section on the electrochemical lactate sensing method, with different lactate concentrations added to the electrochemical electrode surface (0 mM as a baseline, 5 mM, 10 mM, 15 mM, 20 mM, and 25 mM). **Supplementary Fig. 7a** shows the setup of the temperature testing. The sensors were positioned atop a hotplate. Using an infrared

thermometer, the temperature of the electrode-electrolyte interface was measured for the electrophysiological electrode conductivity. The following temperatures were measured: 25 °C, 30 °C, 32.5 °C, 35 °C, 37.5 °C, and 40 °C. **Supplementary Fig. 7b** shows the setup of the humidity testing. The sensors were placed inside a closed acrylic chamber (12 × 12 × 12 inch), along with a mini humidifier inside a beaker of water, a digital temperature and humidity monitor, and fixation helper hands to hold the counter and reference electrodes. Four humidity conditions were measured, including 40%, 50%, 60% and 70% (relative humidity).

Supplementary Fig. 7c,d show the conductivity of the electrophysiological electrodes at various temperatures. Both the impedance and phase of the electrode-PBS interface reveal no significant changes between 25 °C and 40 °C, which corresponds to the normal range of ear temperature. Due to the fact that we were assessing the electrophysiological electrode interface with PBS covering the electrode, the humidity test configuration did not apply to the electrophysiological electrodes. Nonetheless, the stability of the electrophysiological electrodes under varying humidity levels inside the ear can be confirmed by the valid electroencephalogram (EEG) recording following exercise and perspiration production.

Supplementary Fig. 7e–n show the sensors' electrochemical stability characterization results under variable temperature and humidity. Simultaneously with the electrophysiological sensor, the robustness of electrochemical sensors were evaluated for the stability at variable temperature and humidity by performing the sensitivity assessment. Significantly similar sensitivities of the sensors were observed at different temperatures which is attributed by the intact activity of the LOx enzyme and the stable sensor development. Also, the sensitivities at different humidity levels are fairly consistent, which is most likely due to the wet PVA-gel on the top of it which resists the impact of fluctuation of the humidity by marinating the sensing surface wet (100% humidity). **Supplementary Fig. 7e–j** show the dose dependent response of the electrochemical sensor under different temperatures. **Supplementary Fig. 7k–n** shows the dose dependent response of the electrochemical sensor under different humidity levels.

The adhesive at the bottom of the sensors stayed firm, showing no sign of detachment.

Supplementary Note 3 l Characterization of electrophysiological sensing

Electrode-ear impedance equivalent circuit models

The electrode-ear impedance showed a gradual decrease after insertion, which reached a plateau (impedance: <1 MΩ, phase: approximately –21°) after approximately 2 minutes (**Fig. 2a,b**). **Extended Data Fig. 4a–f** reveals granular structure in the surface morphology and the elemental composition of the electrophysiological electrodes after 1 hour of in-ear use. The sodium content of these granules indicated sweat residue, confirming that the decreasing trend in electrode-ear impedance was mostly due to sweat deposition at the electrode-ear contact, converting it from a dry to a wet electrode interface. EIS results in **Fig. 2c,d** show a capacitive to resistive shift of the electrode-ear impedance with decreasing frequency. Such behavior was modeled as an equivalent circuit model comprised of layered conductors and constant phase element (CPE) with an angle <90°.

Several equivalent circuit models have been developed for electrode-skin contact under different conditions[17,25]. The RRC model models the dry contact interface, consisting of a spread resistance R_s, in series to a parallel combination of charge-transfer resistance R_{ct} and double layer capacitance C_{dl}. The RRCRC model models the wet contact interface, adding another RC element to the RRC model to represent the sweat contribution to the interface. The CPE model replaces the C_{dl} with a constant phase element, an imperfect capacitor representing the non-faradaic impedance with a phase shift < 90°. The impedance of a constant phase element is $1/(j\omega)^n Q$, where $0 < n \leq 1$, Q measures the magnitude of Z_{CPE} while n fits the bilayer phase and $n = 1$ represents a capacitor. The total impedance of the three models is listed below:

$$Z_{RRC} = Rs + \frac{R_{ct}}{1 + j\omega C_{dl} R_{ct}}$$

$$Z_{RRC} = Rs + \frac{R_{ct}}{1 + j\omega C_{dl} R_{ct}} + \frac{R_{ct2}}{1 + j\omega C_{dl2} R_{ct2}}$$

$$Z_{CPE} = Rs + \frac{R_{ct}}{1 + (j\omega)^n Q R_{ct}}$$

The equivalent circuit fitting took the averaged electrode-ear EIS data (**Fig. 2c,d**) as the input. Levenberg-Marquard algorithm was used for an amplitude weighing minimization function for the electrode-ear EIS data fitting:

$$r_a(f, P_1, \dots, P_M) = \frac{{r_C}^2}{N - M}$$

$${r_C}^2 = \sum_{i=1}^{N} \frac{(Z_i' - Z_{icalc}')^2 + (Z_i'' - Z_{icalc}'')^2}{{Z_i'}^2 + {Z_i''}^2}$$

Here r_a is the function to be minimized. $N = 50$ is the number of EIS data points across the 1–10 kHz frequency range. M is the number of parameters, $M = 3$ for the RRC and CPE model, $M = 5$ for the RRCRC model. f is the frequency. $P_1 \dots P_M$ are the parameters to be fitted.

Extended Data Fig. 4j–k shows the fitted equivalent circuit models' EIS. The CPE model produced the best modeling accuracy because it can model phase shifts <90°. The RRCRC model has higher accuracy than the RC model for bringing in the wet interface, which corroborates the presence of sweat, as shown in **Extended Data Fig. 4d–f**.

Eye movement signal measurement results and analysis

Two sensing modalities were explored with the in-ear integrated sensors in both ears. Ipsilateral referencing sensing used electrophysiological electrodes and REF electrodes from the same ear, measuring potential differences locally around the ear. Contralateral referencing sensing used electrophysiological electrodes and the REF electrode from the contralateral ear, measuring potential differences across the scalp in a similar manner to the scalp EEG. Due to the transmission attenuation of corneal-retinal signals, local field potentials around the ipsilateral ear are less affected by eye movements. In contrast, contralateral ear potentials are subject to greater eye-motion-induced signal components, as corroborated by a 60-s eye movement sensing session in **Extended Data Fig. 5a,b**, which showed a higher noise floor along with much larger eye movement responses from the contralateral referencing setup. Four common eyeball movements and two eye blinks were characterized with the two referencing configurations, with the averaged time series results from multiple participants shown in **Extended Data Fig. 5c**. Ipsilateral referencing measured 4.84

(±0.31) µV for eyeball movements, including glancing up, down, left, and right, which was 4.60% of the contralateral reference measures of 105.27 (±11.77) µV. Ipsilateral referencing contributed more electromyography (EMG) signals from eyelid movements and measured 30.84 (±2.58) µV for normal or heavy eye blinking, which was 38.89% of the contralateral referencing measures of 79.31 (±3.23) µV. Given that EEG amplitudes are typically less than 30 µV, the results suggest that ipsilateral referencing, or employing both the sensing and reference electrodes in one ear, has better eyeball movement artifact resistance than contralateral referencing, or scalp EEG. Both sensing modalities measured blinking signals, but ipsilateral referencing still recorded a smaller amplitude. According to the findings, ipsilateral referencing is more appropriate for EEG measurements involving eye movements. Contralateral reference is more appropriate for measurements in which the objects of inquiry are eye movement signals.

Electrophysiological sensing validation
The electrophysiological sensing performance of the in-ear integrated sensors was validated by conducting a parallel measurement with a 32-channel dry contact EEG headset (CGX Quick-32, Cognionics) on 2 participants. The setup of the in-ear integrated sensors remained the same as described in the method section. Separately, the EEG headset was mounted over the scalp of the participants (**Extended Data Fig. 7a**). Data transmission was through Bluetooth to the host computer in parallel to the in-ear sensors' data acquisition (DAQ) system. A 500 Hz sampling rate was configured. The impedance of each headset electrode to the skin was monitored prior to the measurement. The acceptable impedance threshold was lower than 2 MΩ, which indicated a solid electrode-to-skin contact. After removing unused ExG and accelerometer channels, the available number of EEG channels was 29. The parallel electrophysiological experiments included the alpha modulation, ASSR, and the eye movement experiments. All the experiments followed the same protocol as those with the in-ear integrated sensors alone. Event markers for the alpha modulation and ASSR experiments were added to the data stream using the EEG headset bundled data acquisition software (CGX Acquisition software, Cognionics).

Alpha modulation: Extended Data Fig. 7c,d show the 2-participant (4 ears in total) average alpha modulation power spectral densities from the in-ear integrated sensors and the EEG headset. Here the grand average results included datasets from all participants' electrophysiological channels. Not all the EEG headset channels recorded significant alpha modulation signals because alpha modulation originates from the specific brain region occipital lobe. 27 channels with the alpha modulation ratio > 1.2 were selected for the grand average calculation. After channel selection, the alpha modulation ratio measured from the EEG headset was R_{AM} = 4.40 (±2.36) (V^2/V^2). By importing the channel location map to the EEGLAB toolbox in Matlab, scalp spectra at 8 Hz, 10 Hz, and 12 Hz were plotted with the locations of headset electrodes. The 10 Hz and 12 Hz spectra clearly showed the alpha modulation activities originated from the occipital lobe, while the 8 Hz spectral drifted to the central frontal region where theta band wave was originated[56]. The same alpha modulation signal was measured with the in-ear integrated sensors as a controlled and parallel experiment. The measured alpha modulation ratios with contralateral and ipsilateral referencing were R_{AM} = 3.38 (±2.01) (V^2/V^2) and R_{AM} = 2.41 (±0.31) (V^2/V^2), respectively. These results show that the EEG headset obtained a higher amplitude modulation ratio, due to the more than 9 times more channels utilized than the in-ear integrated sensors. Some channels from the EEG headset were close to the source of alpha modulation and produced a much higher amplitude modulation ratio (e.g., channel O1 located around the occipital lobe got a R_{AM} = 10.63). With the contralateral referencing setup, the in-ear integrated sensors practically measured the potential difference across the scalp, which was comparable to that of the EEG headset. This can be demonstrated by the similar noise floor (described as power spectral density (PSD) at 15–20 Hz in **Extended Data Fig. 7c,d,** both 2–4 dB (rel. to µV/√Hz)) from the EEG headset and the contralateral referencing in-ear measurements. In-ear measurements using ipsilateral referencing had the lowest amplitude modulation ratio, which was consistent with the fact that ipsilateral referencing assessed mostly the local potential field surrounding the ear.

Auditory steady-state response: Extended Data Fig. 7e,f show the 2-participant (4 ears in total) average 40- Hz ASSR power spectral densities from the in-ear integrated sensors and the EEG headset. Here the grand average results included datasets from all participants' electrophysiological channels. Not all the EEG headset channels recorded significant ASSR signals because ASSR originates from the specific brain region, the temporal auditory cortices. 19 channels with the ASSR signal to noise ratio (SNR) > 1.5 were selected for the grand average calculation. After channel selection, the ASSR SNR measured from the EEG headset was 3.70 (±2.36) dB. By importing the channel location map to the EEGLAB toolbox in Matlab, the 40 Hz scalp spectra were plotted with headset electrodes' locations, which showed the auditory responses originated from the temporal auditory cortices. The same ASSR signal was measured with the in-ear integrated sensors in a controlled and parallel experiment. The ASSR SNR obtained with ipsilateral referencing was 7.38 (±1.72

dB). While the ASSR signal was not observed with contralateral referencing. These results show that the ipsilateral referencing in-ear measurements obtained a higher ASSR SNR due to proximity to the auditory cortices. Nevertheless, channels of the EEG headset close to the auditory cortex also produced a significant ASSR (e.g., channel CP6 located around the right auditory cortex got an ASSR SNR of 10.63 dB). Contralateral referencing in-ear measurements were comparable to the EEG headset with a comparable noise floor (described as PSD at 40–80 Hz in **Extended Data Fig. 7e,f,** both −2 to −4 dB (rel. to µV/√Hz)). In-ear Ipsilateral referencing outperformed contralateral referencing regarding ASSR measurements.

Eye movement: Extended Data Fig. 7b shows the synchronous eye movement signals measured by the in-ear integrated sensors and the EEG headset. For eyeball movements including glancing up, glancing down, glancing left, and glancing right, the EEG headset recorded an averaged amplitude of 175 µV, 129 µV, 124 µV, and 151 µV, respectively, which accounted for a grand average amplitude of 144.75 µV. And for heavy eye blinks and normal eye blinks, the EEG headset recorded an average amplitude of 994 µV and 276 µV, which accounted for a grand average amplitude of 635 µV. The EEG headset recorded a significantly larger amplitude when comparing the eye movement signals measured from the in-ear integrated sensors and the dry contact EEG headset. For eyeball movements, the headset measured an amplitude 29 times higher than the ipsilateral referencing measurements and 130% higher than the contralateral referencing measurements. For eye blinking, the headset measured an amplitude 20 times higher than the ipsilateral referencing measurements and 8 times higher than in-ear contralateral referencing measurements. Apart from the instructed 6 eye movements, the EEG headset also recorded more EMG artifacts as shown at t = 35.78 s, 62.87 s, 62.07 s, 75.46 s, and 84.14 s, which were not observed from the in-ear measurements. In summary, in-ear ipsilateral referencing measurements recorded the smallest eye movement signals and practically no eyeball movement signals. In-ear contralateral referencing measurement recorded both the eyeball movements and eye blinking. However, its amplitude was much less than the dry contact EEG headset, which had the highest sensitivity and recorded more EMG artifacts.

Simultaneous electrophysiological sensing

The Integrated in-ear sensors are capable of simultaneous sensing of multiplexed electrophysiological modalities. Particularly, electrodermal activity (EDA), electrooculography (EOG), and EEG features including alpha, ASSR reported in **Fig. 2** can be measured at the same time with the same electrophysiological sensors. This multiplexing of electrophysiological signals in the ear allows for a more complete knowledge of physiological processes that occur throughout different cognitive and emotional states. EEG can offer information on cognitive processes, such as attention and arousal, and assist with the identification of neurological illnesses by measuring electrical activity in the brain. EOG detects electrical activity in the muscles that regulate eye movements and can offer information regarding eye movements during a variety of cognitive activities, including reading and visual search. And EDA monitors the electrical conductance of the skin and can offer information about the activity of the sympathetic nervous system, which is related to emotional arousal and stress[57].

Baseline: To demonstrate the feasibility of electrophysiological sensing multiplexing, a number of experiments were conducted on one participant using the ipsilateral referencing setup to simultaneously measure EEG and other electrophysiological modalities. First, a baseline experiment was conducted to evaluate solely the EEG-related 40-Hz ASSR response. Using the same experimental setup as outlined in "Methods: electrophysiological measurement system integration and the on-body setup", the electrophysiological signals were sampled at 500 Hz. The participant was presented with 40-Hz ASSR stimuli for one minute, during which time the participant's eyes remained closed. **Extended Data Fig. 6a,b** shows the baseline EEG streaming data in 1 minute and the obtained 40 Hz ASSR SNR to be 15.28 (±4.00) dB.

EEG + EOG: The purpose of the combined EEG and EOG experiment was to show the measurement of eye movements, including eye blinks, while simultaneously collecting characteristic EEG features. As indicated in **Extended Data Fig. 5**, ipsilateral referencing captured eye blink signals with a much lower amplitude than the contralateral referencing configuration (and also EEG headset). The measurement was conducted in the same manner as the baseline experiment, with the participant instructed to blink every five seconds. **Extended Data Fig. 6c** shows eye blinking waveforms with a <100 µV, 5-s interval that are similar with the ipsilateral referencing EOG results in **Extended Data Fig. 5**. **Extended Data Fig. 6d** shows the PSD of the 1-minute EEG and EOG measurements with a 40-Hz ASSR SNR to be 17.36 (±2.86) dB. The result demonstrates simultaneous EEG and EOG data streaming, with EOG artifacts having negligible impact on EEG feature extractions. In this experiment, the EOG artifacts increased the <10 Hz band power by 7.40 dB. Particularly, EOG artifacts are generally low-frequency signals (<10 Hz) that may overlap with EEG activity in

this frequency range. This may lead to erroneous identification of low-frequency EEG activity, although the 40 Hz ASSR characteristic in this example remained unaffected, as corroborated by the ASSR SNR.

EEG + EDA: The purpose of the combined EEG and EDA experiment was to show the measurement of ear-electrode impedance while simultaneously recording EEG-specific characteristics. As introduced in the Methods section, the electrophysiological electrodes #1, #2, and #3 were connected to a potentiostat as WE, RE, and CE while still being connected to the electrophysiological DAQ system. The impedance was measured by applying 100 nA of sinusoidally alternating current between the WE and CE while monitoring the voltage drop between the WE and RE. Continuous impedance measurements were performed at three frequencies: 10 Hz, 100 Hz, and 500 Hz. **Extended Data Fig. 6e,g,i** show the simultaneous streaming of EEG and EDA data. EEG stream from electrophysiological sensor #2 was presented here since it only served as the RE in the impedance measurement and did not carry any current. The existence of periodic sinusoidal waveforms indicates current injection for impedance measurement. Measuring impedance at a higher frequency reduces artifacts in both the temporal and frequency domains of the EEG signal. This is shown by the decreasing amplitude of sinusoidal waveforms in the zoomed-in insets of **Extended Data Fig. 6e,g,i**, which occurred due to the stray capacitance in the potentiostat I/E converter (transimpedance amplifier) forming a low-pass RC-filter and also the capacitive component in the electrode-ear interface. In addition, since the majority of EEG-related frequency characteristics are less than 100 Hz, it is advantageous to perform impedance measurements for applications such as sweat analysis and lead off detection at a higher frequency band, as impedance measurements at lower frequencies do not carry more meaningful information. **Extended Data Fig. 6f,h,j** show 40-Hz ASSR SNR with 10 Hz, 100 Hz, and 500 Hz concurrent impedance measurement to be 11.37 (±1.62) dB, 11.07 (±1.02) dB, and 16.25 (±0.55) dB, respectively. Here **Extended Data Fig. 6e** shows a peak around 10 Hz resulted from the 10 Hz impedance measurement, while **Extended Data Fig. 6j** demonstrates that the out-of-band (500 Hz sampling frequency) 500 Hz impedance measurement has negligible influence on the EEG PSD, as evidenced by a similar 40-Hz ASSR to the baseline result.

EEG Motion artifact reduction

EEG data collected from participants in motion is susceptible to degradation due to motion-induced artifacts. Motion artifacts are primarily attributable to two sources, first is charge buildup at the ear-electrode interface from motion-induced displacement of electrodes relative to the ear canal, and second is cable movement, altering electrical characteristics at the contact site, and generating electromagnetic interference that could be picked up by the electrodes[58].

This study's experiments included both extremes of motion-artifact induction, where participants either engaged in intense physical activity (as with the combined EEG and lactate sensing experiment), or were instructed to remain motionless (for data segments used for alpha modulation and cognitive state analyses). Artifact rejection was performed by visual examination of EEG data and manual rejection of motion-related segments.

Removing motion artifacts is a challenging topic in electrophysiological research, usually requiring hardware and software solutions. Previous research[59] has demonstrated the efficacy of various signal processing methods for reducing motion artifacts, including independent component analysis (ICA), common average referencing, adaptive filtering, spectral (wavelet) decomposition, and automatic subspace reconstruction (ASR)[60]. These signal processing techniques could be applicable to recordings acquired from the in-ear integrated sensors used in this study, and here we investigate the feasibility of ASR for reducing[61] motion artifacts in in-ear EEG applications.

ASR is an adaptive spatial filtering technique that can be run online to remove high amplitude data components, relative to an artifact-free reference window that could be collected as a short (1 minute) calibration period[62].This method performs a series of linear subspace projections on a sliding window of data to identify and remove principal components exceeding spatially varying thresholds determined during calibration. The removed components are interpolated and projected back to channel space. ASR provides tunable parameters including a cutoff parameter for adjusting channel variance thresholds that decide when ASR corrections would apply, and a window length of data for computing channel variances, which is expected to be on a similar timescale as the artifacts to be removed. ASR is available as part of the clean_rawdata plugin in EEGLAB.

The following experiment was performed to investigate the feasibility of using ASR with this study's in-ear system: a participant wearing the in-ear integrated sensors was instructed to stand motionless for about 1

minute, followed by a minute of uncued feet stamping. The participant was asked to keep their eyes closed for this entire duration, while listening to a 40 Hz ASSR stimulus generated and delivered as described previously. The recorded data was imported into MNE-Python, and bandpass filtered with cutoffs 0.1 and 50 Hz. The filtered time series for the entire experiment is shown in **Supplementary Fig. 8a**, with boxes identifying two manually selected 1 minute time windows corresponding to the baseline (minimal artifacts) and motion (stamping) segments. The baseline segment (zoomed in **Supplementary Fig. 8b** was aligned with the start of the experiment, and the motion segment (zoomed in **Supplementary Fig. 8d**) was selected to be a few seconds into the stamping activity, to prevent the relatively smaller motion artifacts from the commencement of physical activity from favoring ASR evaluation.

The filtered data was then imported into EEGLAB to run ASR calibration on the baseline segment using the asr_calibrate function with the cutoff parameter set to its default value of 5, and a reduced window length (window_len) of 0.1, to allow multiple windows to fit within one stamping cycle. Subsequently, the motion segment was filtered using the asr_process function (**Supplementary Fig. 8e**). PSDs were calculated for all three channels (EEGLAB's spectopo function, using a 10-s time window with no overlap), linearized, averaged, and converted back to log scale to get the channel mean shown in **Supplementary Fig. 8c,f**. The 40 Hz ASSR peak and the 31.2 Hz (for lead-off detection) impedance measurement peaks are identifiable for the baseline period (**Supplementary Fig. 8c**, but become occluded by the elevated noise floor during stamping motion (**Supplementary Fig. 8f**). Running ASR is able to push the noise floor down such as the ASSR and impedance measurement peaks become visible again. Comprehensive hardware and software design considerations should be included for better motion artifact rejection, for example, an immediate future direction for this work is to further integrate the in-ear sensors with the backend DAQ system to eliminate dangling wires.

Supplementary Note 4 | Characterization of the combined electrophysiological and electrochemical sensing crosstalk

EEG sensing to lactate sensing crosstalk analysis
Since EEG measurement was a passive potentiometric measurement, its effects on the lactate sensing were expected to be negligible. Under uncommon circumstances, the EEG sensors may be disconnected, resulting in transient charge transfers that may affect the lactate CA current measurements. Besides, for common-mode rejection, the driven right leg (DRL) electrode used in the EEG measurement sensed the common-mode voltage from the 3 electrophysiological electrodes and actively drove the concha cavum with an inverted common-mode signal to create a negative feedback loop. This driven signal could potentially propagate to the electrochemical electrodes and affect the lactate sensing results. A lactate sensing environment was created by drop casting 10 μL 0.1 mol/L PBS with 5 mmol/L Lactate to the PVA hydrogel to characterize these effects. A 180-s lactate sensing CA measurement was taken on two participants with a synchronous EEG measurement session. During this period, the following changes were made to the EEG measurement setup: physically disconnecting electrophysiological sensors from the DAQ, reconnecting electrophysiological sensors to the DAQ, disconnecting the DRL sensor from the DAQ, and powering off the DAQ.

Supplementary Fig. 10 shows the synchronous lactate CA current and EEG waveforms. First, the steady-state lactate CA current was approximately –1.9 µA, larger than those in **Extended Data Fig. 9e,h** because of the presence of lactate in the PVA hydrogel. Disconnecting electrophysiological sensors resulted in the saturation of corresponding electrophysiological sensors and a ~0.3 s, <50 nA fluctuation of lactate sensing current. Disconnecting DRL resulted in more noise in the EEG waveform but had a negligible effect on the lactate CA current. Powering off the DAQ also did not have a discernible effect on the lactate CA current. In conclusion, since the lactate sensing relies on the steady-state CA current, none of the EEG actions resulted in interference with the lactate sensing results.

Lactate sensing to EEG sensing crosstalk analysis
Major crosstalk was expected from the lactate measurement to the EEG measurement since the lactate CA measurements required applying a –0.2 V potential between the WE and RE, which may drift the nearby electrophysiological sensor recordings (**Extended Data Fig. 9a**). Several design considerations were made to minimize such potential crosstalk. An insulation SEBS layer was first built to insulate all the electrodes. The electrophysiological 3D electrodes and electrochemical electrodes had a ~750 µm height difference, which was made up by the height of the PVA hydrogel covering the electrochemical electrodes, serving as a sweat collector. Due to the existence of the PVA hydrogel, which produced a low-impedance pathway under a sweaty condition, most of the lactate CA current would flow through the PVA hydrogel instead of the skin, which could affect the EEG measurements.

Such potential crosstalk effects from the lactate measurement to the EEG measurement were evaluated by recording the corresponding signals during on-body operations. The experiment considered two factors. The first factor was the sweat in the ear, which affected the conductivity of the ear canal, the electrode-ear interface, and the PVA hydrogel. After cleaning the participants' ears with alcohol pads before the experiment, both a less sweaty and a sweaty environment were simulated by drop casting deionized (DI) water (**Extended Data Fig. 9b**) or saline (**Extended Data Fig. 9c**) to the PVA hydrogel covering the electrochemical sensor. The second factor was the electrochemical CA setup. By design, EEG measurements permit continuous monitoring of cognitive brain state for BCI applications, throughout extended time periods of wearable use. In contrast, lactate measurement produces an intermittent signal for fitness applications, at times when sweat is being generated. Following these two factors, under both the less sweaty and the sweaty conditions, the co-sensing crosstalk experiments consisted of a 70-s 40-Hz ASSR measurement session, followed by a 60-s electrochemical CA measurement session starting at 5 s after the onset of the EEG session and ending at 5 s before the EEG session ended. Before the crosstalk experiment, a 70-s ASSR baseline experiment was first conducted without any CA measurement session in parallel. **Extended Data Fig. 9d** shows the 3-participant-averaged baseline 40 Hz ASSR PSD, with an 18.72 (±1.40) dB SNR.

Extended Data Fig. 9e,f and **Extended Data Fig. 9h,i** show the 3-trial-averaged synchronous CA and EEG waveforms in the less sweaty condition and sweaty condition, respectively. The electrochemical electrodes measured steady-state currents of –0.25 µA and –0.39 µA under the two conditions, with their difference due to the conductive saline utilized in the sweaty condition. The –0.2 V potential was applied at the onset of the lactate sensing session (t = 5 s), resulting in a transient artifact and a discharging phase to the EEG data

stream. For electrophysiological sensors 1 to 3, the resulting transient artifact had an amplitude of −17.7 mV, −10.2 mV, and −1.9 mV under less sweaty conditions, and a more prominent amplitude of −75.4 mV, −33.2 mV, and −2.1 mV under sweaty conditions. This amplitude distribution revealed a location-dependent relationship: the closer the electrophysiological electrode was to the electrochemical electrodes, the greater the amplitude of the transient artifact. From the observation of the waveform, it took approximately 5 s (less sweaty condition) and 7 s (sweaty condition) for all the three electrophysiological sensors to return to their pre-artifact state (**Supplementary Fig. 9a,f**). From the spectral analyses (**Supplementary Fig. 9b–e** and **Supplementary Fig. 9g–j**), the transient artifacts, which had a broadband frequency spectrum, elevated the noise floor of the ASSR PSD and corrupted the corresponding 40-Hz ASSR peak. The artifact blanking method was used to remove the artifact. Here a period of the EEG time series data was rejected for EEG analyses (**Extended Data Fig. 9e,h** red shaded region represents the data rejection period; the blue shaded region represents the data acceptance period). After a 3-trial-averaged 9.63-s data rejection (less sweaty condition), and a 3-trial-averaged 10.78-s data rejection period (sweaty condition), **Extended Data Fig. 9g** and **Extended Data Fig. 9j** shows a corrected ASSR PSD (as derived in Supplementary Text Note 4) comparable to the EEG-only baseline result (**Extended Data Fig. 9d**) with an SNR of 18.63 (±0.84) dB and 19.69 (±0.81) dB, respectively**.**

Spearman correlation analysis was used to determine the correlation between the corrected co-sensing ASSR PSD results obtained using the artifact blanking method and the baseline ASSR PSD results. Between the EEG-only baseline and the corrected ASSR, the Spearman's ρ-values were 0.957 and 0.950 in the less sweaty and sweaty conditions, respectively. These findings reveal that after removing the transient artifact from the EEG data stream, the spectral analyses of the EEG readings were hardly affected by the electrochemical CA measurement.

Spectral analyses of the EEG with chronoamperometric induced artifacts
The co-sensing crosstalk results showed that a transient artifact and a discharging phase were introduced to the EEG measurement at the start of the CA measurement. Since the transient artifact, or in this case, an impulse, was a constant value across all frequencies between 0 to $fs/2$ Hz, the PSD of the corresponding EEG signal will have an elevated noise floor. The artifact blanking method was used to remove such artifacts. As shown in **Extended Data Fig. 9e,h**, a portion of the EEG time series data was rejected for analyses after the onset of the transient artifact. This section analyzes how different selection of this data rejection period affects the final EEG spectral analyses.
To investigate the noise performance of the artifact, unlike the methods used for alpha modulation or ASSR characterizations, no time-domain averaging (resulting in a smoothed noise floor) was used here for power spectral density calculation, and a longer window length of 50 s was used to achieve higher frequency resolution. The calculated PSD was shown in the log-log scale to demonstrate the 1/f noise common for biological systems. Four EEG data rejection periods were selected:

1. No data rejection: the whole 70-s EEG data stream was used to analyze the artifact's full effect on the EEG PSD.
2. 15 ms data rejection period: the EEG data stream 15 ms after the onset of the electrochemical CA measurement included the data stream after the peak of the transient artifact, analyzing the effect of the discharging phase.
3. 1 s data rejection period: an intermediate stage.
4. 5 s (less sweaty condition) or 7 s (sweaty condition) data rejection period: after investigating the waveforms (**Extended Data Fig. 9e,h**), the whole transient artifact along with the discharging phase was rejected for EEG analyses.

Supplementary Fig. 9b–e and **Supplementary Fig. 9g–j** show the PSDs calculated with the abovementioned four data rejection periods in the less sweaty and sweaty conditions. The results showed the artifact's location dependence, which was also shown by the amplitude of the transient artifact itself. With the increase of the EEG data rejection period, the elevated noise floor of the three sensors gradually decreased to the same level (Also indicated by the decrease of 1/f noise corner frequency). The artifact in the sweaty condition brought in more interference than the less sweaty condition, which was indicated by the elevated noise floor and the longer data rejection period needed to remove the artifact. After 5 s in the less sweaty condition, or 7 s in the sweaty condition, the discharging phase of the artifact appeared to end from the time domain, and the PSDs of the three electrophysiological sensors almost overlapped from the frequency domain.

Data rejection period calculation: As a quantitative and more accurate approach, the length of the data

rejection period was further determined by an iteration approach. For each of the 3 crosstalk EEG measurement datasets, the data rejection length increased by 0.1 s in each iteration. The resulting EEG PSD was correlated with its EEG-only baseline EEG PSD until it first reached a >0.95 Spearman's *p*-value. As a result, data rejection periods of 8.9 s, 11.2 s, and 8.8 s (9.63 s on average) were required for the three EEG datasets in the less sweating condition. The required data rejection times for the sweating condition were 10.5 s, 11 s, and 10.8 s (10.78 s on average). Using the averaged data rejection periods, the corrected 40 Hz ASSR PSDs are shown in **Extended Data Fig. 9g,j.** The resulting PSDs in the co-sensing experiments were comparable to the EEG-only baseline results (Spearman's *p*-value > 0.95) with close ASSR SNRs. In conclusion, with a conservative data rejection period of 11 s, the artifact resulting from the electrochemical CA measurement will not affect the EEG spectral analyses results.

Supplementary Note 5 | Evaluation of usability and wearability

Usability and wearability of the in-ear integrated sensor have been evaluated as a key design objective. As previously stated, questionnaires and interviews are presently the most common methods for assessing donning comfort[63]. In this study, questionnaires were used to evaluate the in-ear integrated sensors. Based on the comfort rating scales from a previous study on wearability evaluation[64], 50 participants were selected at random, primarily from the campus of the University of California, San Diego. Questionnaires were created using Google Survey. Each participant was instructed to examine the questions thoroughly before evaluating the sensors on the body using any method they deemed appropriate.
Detailed questionary questions and their rating scales were defined as follows. Each question was designed to evaluate one category of usability and wearability. As a general principle, a higher score from each question indicates more usability and wearability.

- **Emotion**: "I am worried about how I look when I wear the sensors. I feel tense or on edge because I am wearing the sensors". A linear rating scale from 1 to 5 represents "very worried" to "very comfortable", respectively.
- **Attachment**: "I can feel the device on my body. I can feel the device moving". A linear rating scale from 1 to 5 represents "I can feel the sensors strongly" to "I cannot feel the sensors at all".
- **Harm**: "the device is causing me some harm. The device is painful to wear." A linear rating scale from 1 to 5 represents "very harmful" to "not harmful at all".
- **Perceived change**: "wearing the device makes me feel physically different. I feel strange wearing the device". A linear rating scale from 1 to 5 represents "considerable change perceived" to "no change perceived".
- **Movement: "**the device affects the way I move. The device inhibits or restricts my movement". A linear rating scale from 1 to 5 represents "notably affected" to "not affected"
- **Anxiety:** "I do not feel secure wearing the device". A linear rating scale from 1 to 5 represents "very not secure" to "very secure".

Supplementary Fig. 13 shows the results of the questionnaires. The in-ear integrated sensors in this work recorded 4.25 points for "Emotion", 4.18 points for "Attachment", 4.35 points for "Harm", 4.16 points for "Perceived change", 4.33 points for "Movement", and 4.20 points for "Anxiety". All the scores reported were weight-average based on the count of responses at each rating. All six usability and wearability categories averaged a score greater than 4, indicating that participants were overall satisfied (>80% rating percentile) with the usability and wearability of the sensors described in this study.

Supplementary Figures

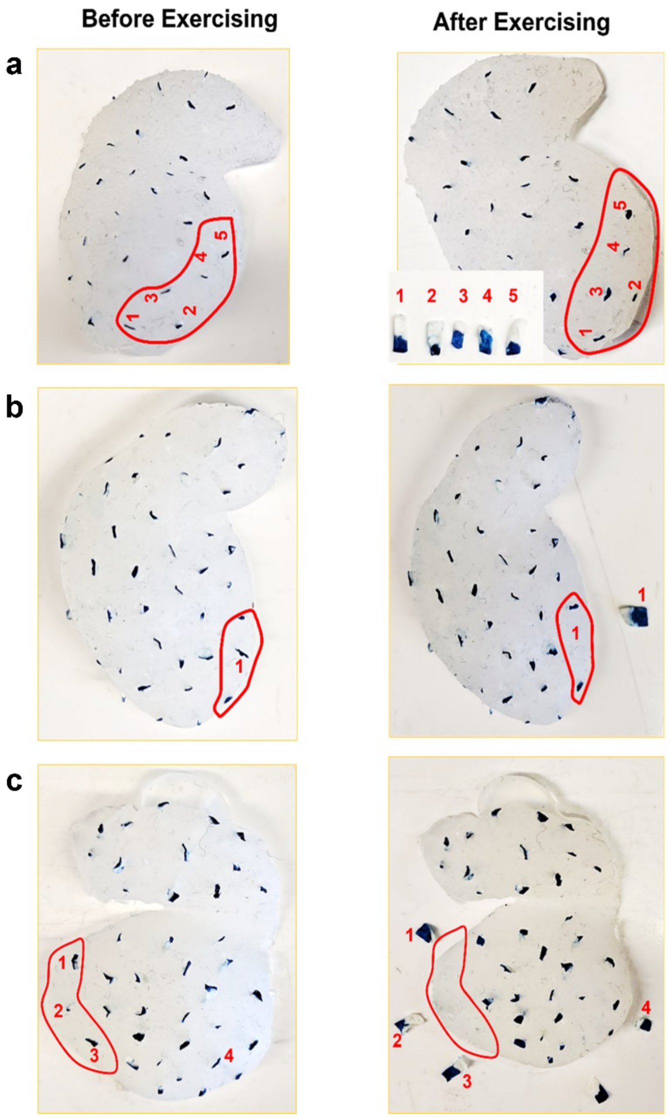

Supplementary Fig. 1 l Sweat profiles on 3 different participants. a–c. The spreading of commercial dye deposited on traces of filter paper pieces was evaluated on 3 healthy participants (n = 3). The left side of the figure presents the filter paper pieces before 30 minutes of exercise. The right side of the figure shows the spreading of the blue dye after 30 minutes of exercise.

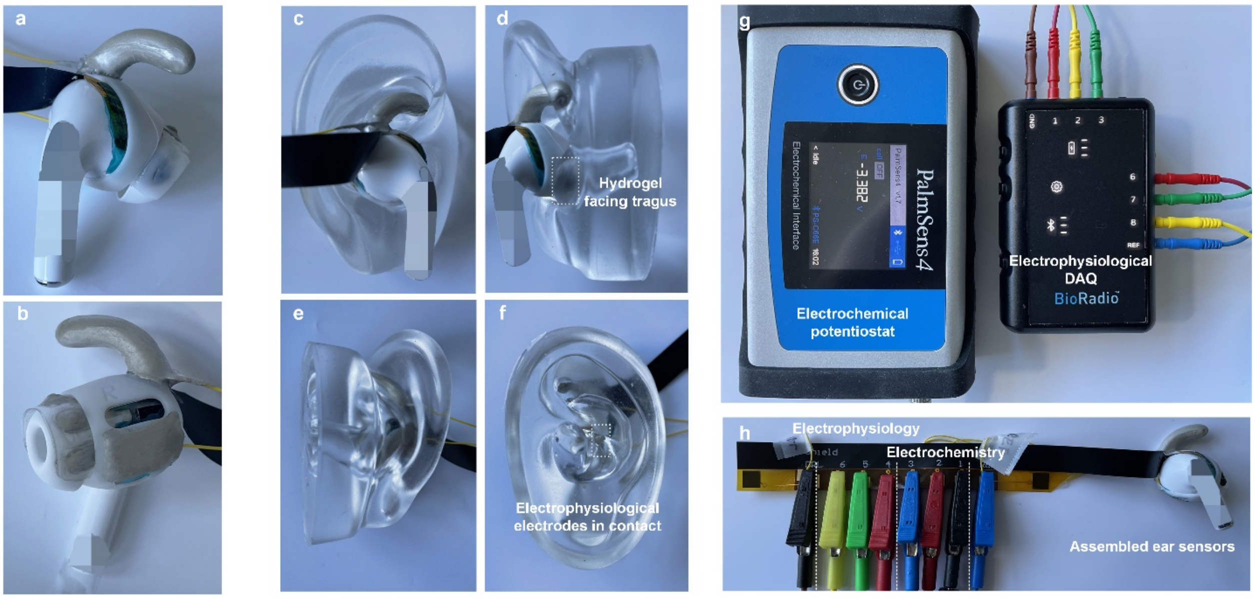

Supplementary Fig. 2 l Assembled in-ear integrated sensors, insertion demonstration, and backend electronic connections. a–b. Images of assembled in-ear integrated sensors on the earphone from different angles. **c–f.** Images of the in-ear integrated sensors inserted into an ear phantom from different angles. **g.** Instrumentations used for electrophysiological and electrochemical signal acquisition. **h.** Connection of ear sensors fPCB to the backend acquisition systems.

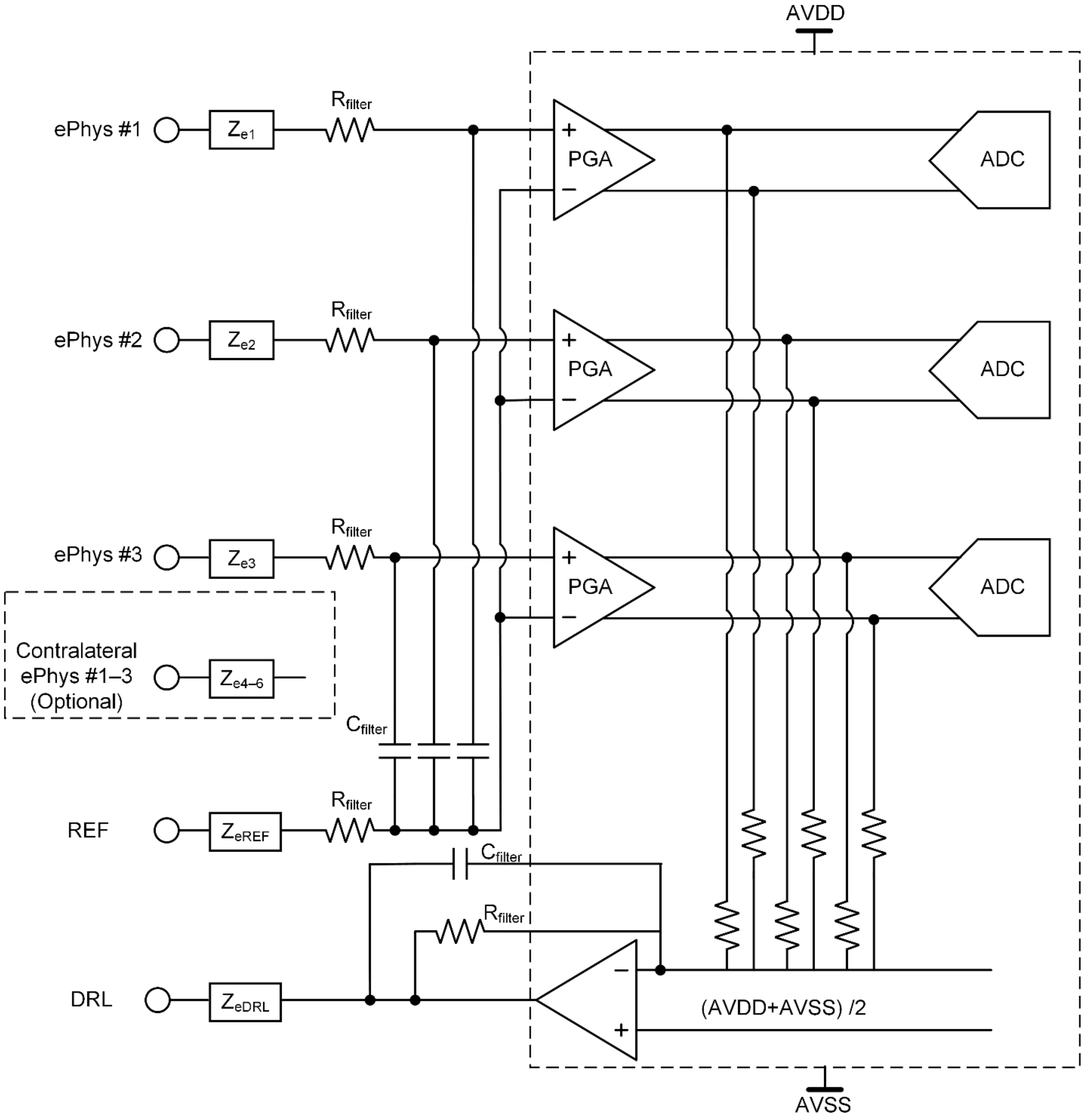

Supplementary Fig. 3 | Electrophysiological front-end circuitry. The single-ended mode was used across all electrophysiological sensors, which uses a common REF. DRL was used to suppress common-mode interference (Component abbreviations in the figure: analog digital converter (ADC), programmable gain amplifier (PGA), analog positive power source to the metal–oxide–semiconductor field-effect transistor (MOSFET) drain (AVDD), analog negative power source to the MOSFET source (AVSS).

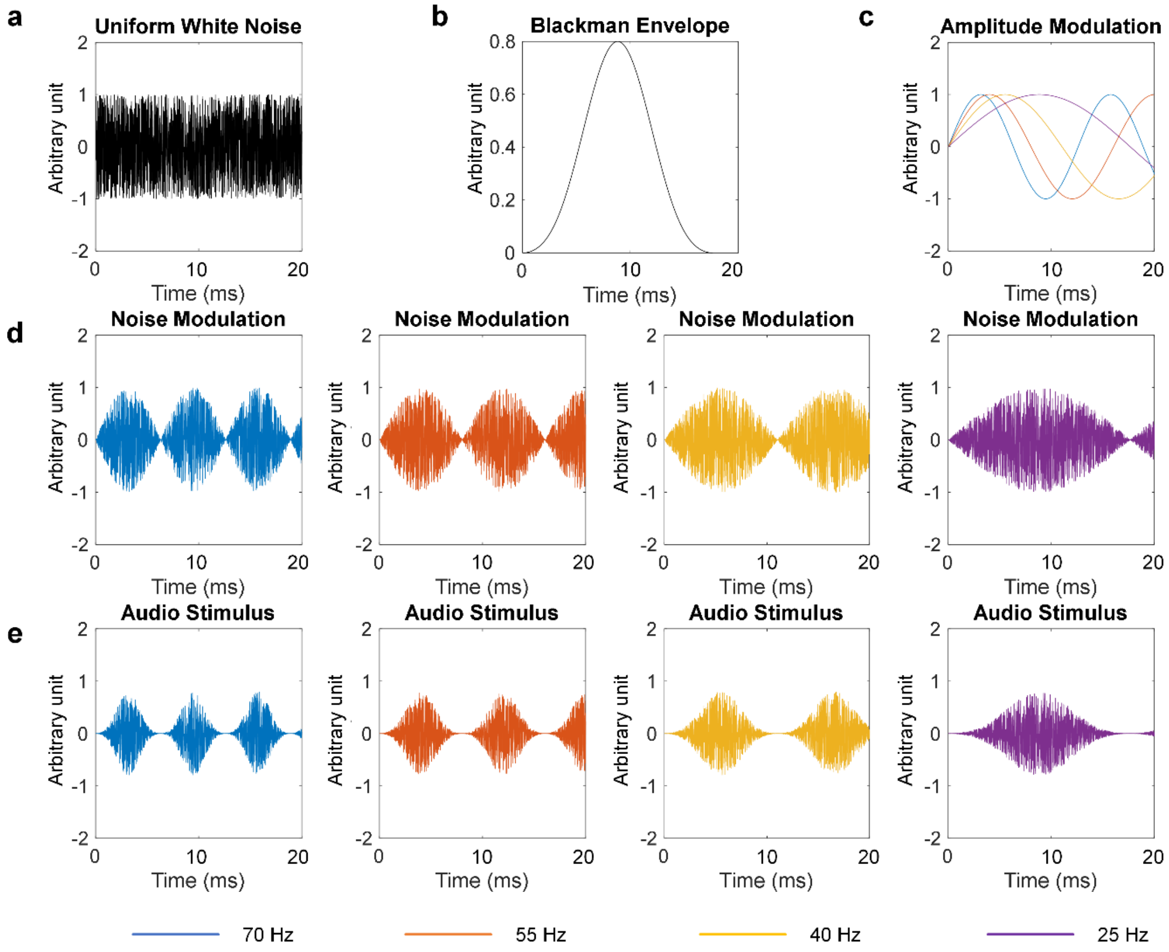

Supplementary Fig. 4 I ASSR stimulus generation. a. Uniformly distributed white noise. **b.** Blackman envelop. **c.** A set of carrier sinusoidal signals (25 Hz, 40 Hz, 55 Hz, 70 Hz). **d.** White noise is convolved with the carrier signals to produce amplitude modulated signals. **e.** A Blackman envelop, as shown in (**b**) is applied to the separated waveforms, producing an appropriately shaped and timed ASSR sound stimulus.

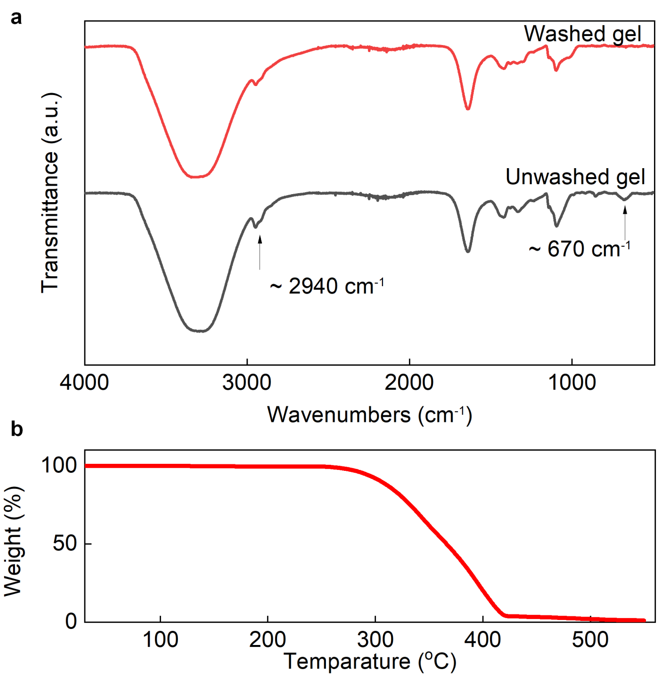

Supplementary Fig. 5 | Characterization of interfacing gel and sensing patch for skin irritants and allergic materials. a. PVA gel characterization for the presence of KOH. FTIR spectrums have been recorded before and after washing the gel. The peak at ~670 cm^{-1} in the spectrum (before wash; Black spectrum), is attributed to the bending vibration of K-O bonds in the KOH crystals disappearing after washed gel (red spectrum), indicating the absence of KOH in the washed PVA gel. **b**. SEBS film characterization for the presence of toluene. The TGA curve demonstrates the mass loss at different temperatures. The consistent weight till 250 ^{o}C indicated the SEBS film is devoid of the toluene which evaporated at the boiling point (~110 ^{o}C). The decrease in weight starting from ~250 ^{o}C is attributed to the heat degradation of SEBS.

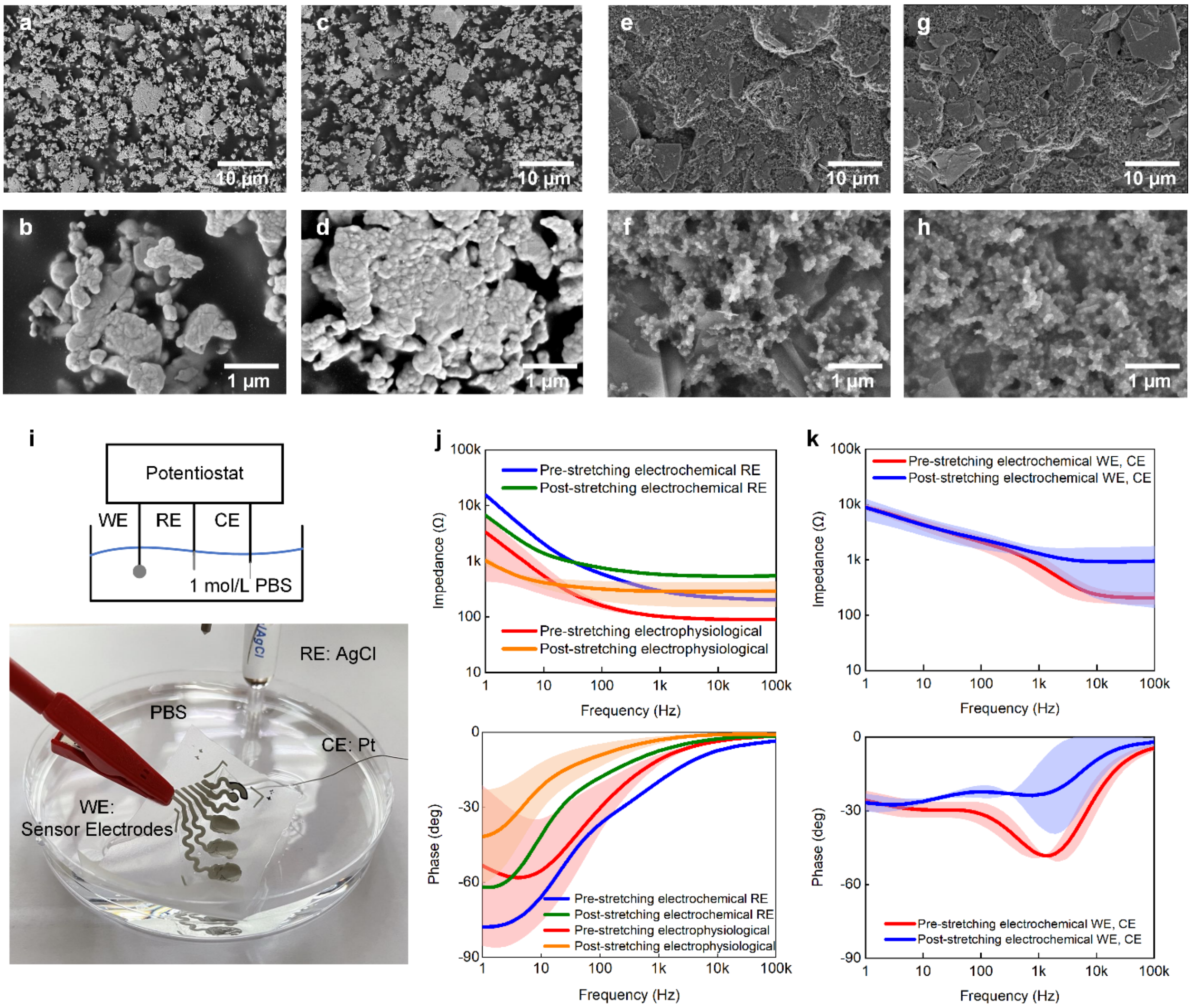

Supplementary Fig. 6 | Mechanical stability testing results of the in-ear electrodes. a–h. SEM of the in-ear integrated sensor electrodes before and after mechanical stretching of 100 cycles. (**a**–**d**) SEM of the Ag-type Electrodes, (**a**,**b**) before and (**c**,**d**) after 100 mechanical stretching cycles. (**e**–**h**) SEM of the PB-type electrodes, (**e**,**f**). before and (**g**,**h**). after 100 mechanical stretching cycles. **i–k.** Impedance test of the in-ear integrated sensor electrodes before and after mechanical stretching of 100 cycles. (**i**) Electrochemical impedance spectroscopy setup in 1 mol/L PBS solution. (**j**) Averaged impedance of the Ag/AgCl surfaced electrodes before and after performing 100 cycles of horizontal and longitudinal stretching. (**k**) Averaged impedance of PB surfaced electrodes before and after performing 100 cycles of horizontal and longitudinal stretching. For all the averaged impedance results, the means are shown as solid lines, while the standard deviations are shown as shaded bands.

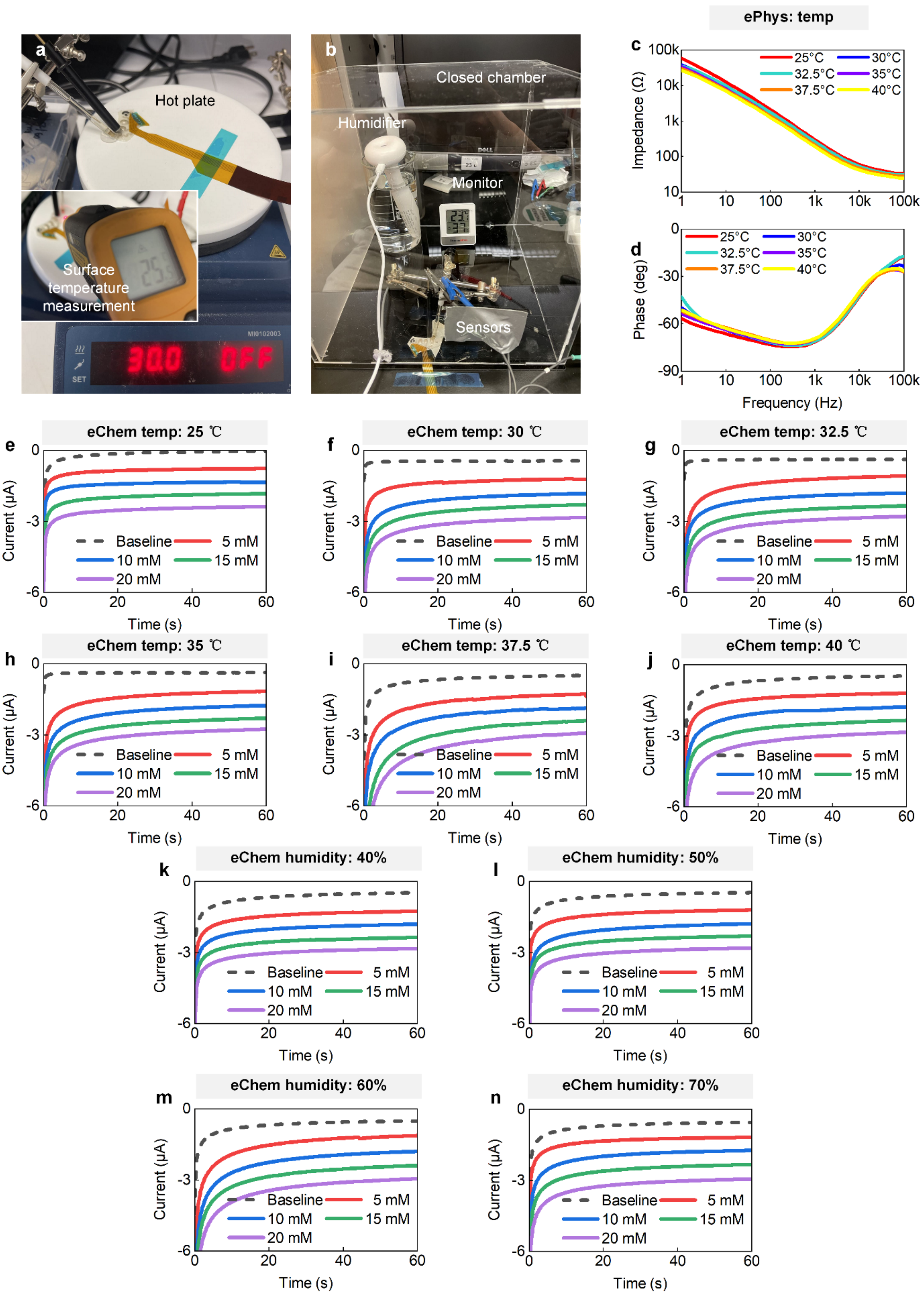

Supplementary Fig. 7 | Electrophysiological and electrochemical sensors stability test results under different temperature and humidity conditions. a. Temperature test setup with the sensors placed on the hotplate, while the sensor's surface temperature was measured with an infrared thermometer. **b.** Humidity test setup with a humidifier, a humidity monitor, the sensors, and fixation helper hands placed in a closed chamber. **c.** Impedance and **d.** phase measurements of the electrophysiological electrodes under different temperatures by drop casting 0.1 M PBS (pH-7.3) onto the electrode surface and a 3-electrode EIS setup. **e**–

j. Sensitivity testing of the electrochemical electrodes under different temperatures with different concentrations of lactate added on. **k**–**n.** Sensitivity testing of the electrochemical electrodes under different humidities with different concentrations of lactate added on. Here the temperature conditions were chosen as 25 °C, 30 °C, 32.5 °C, 35 °C, 37.5 °C, and 40 °C, respectively. The humidity conditions were chosen as 40%, 50%, 60% and 70% (relative humidity), respectively.

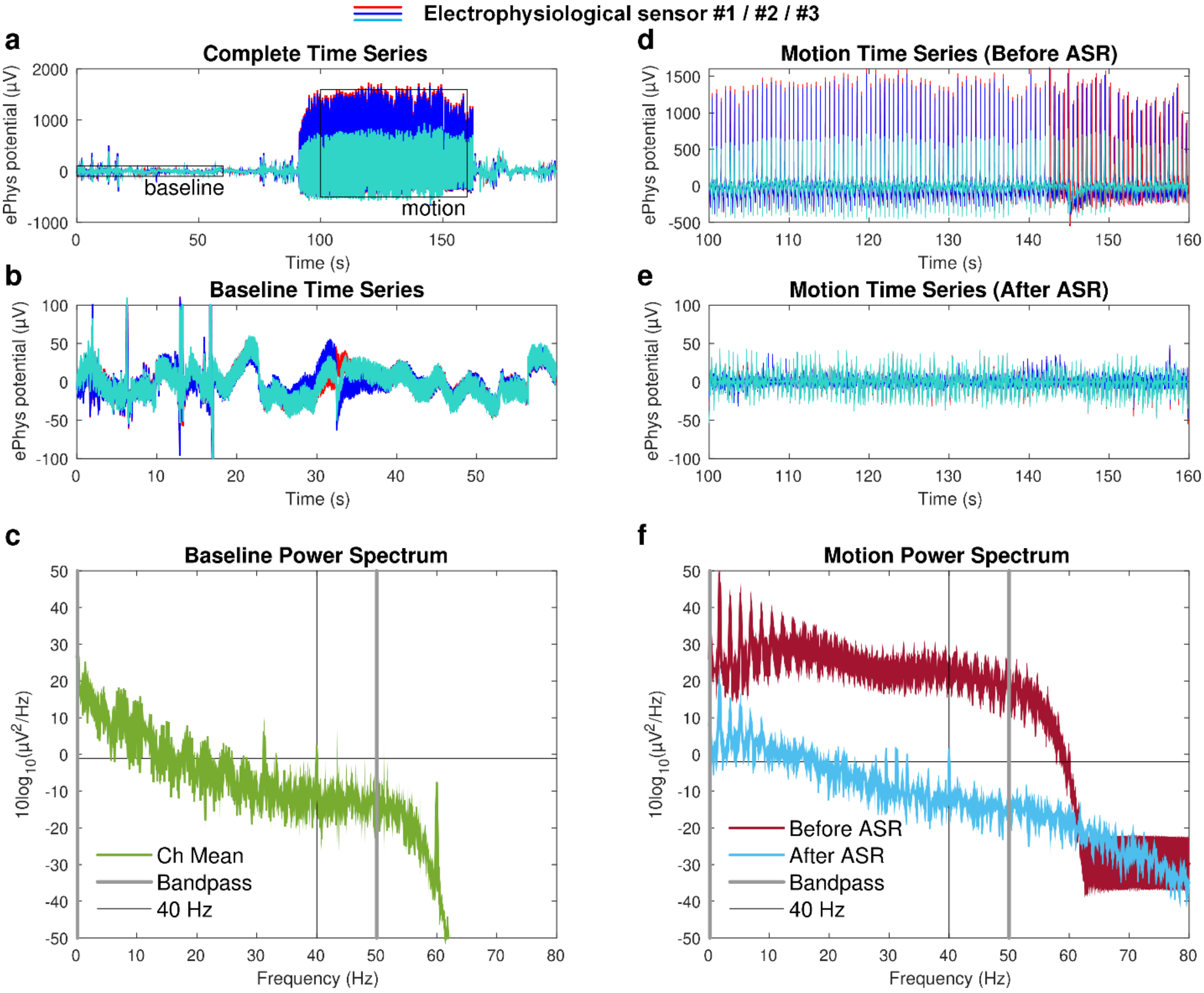

Supplementary Fig. 8 I Motion artifact reduction analysis for the in-ear electrophysiological sensors. **a.** Bandpass filtered time series for the stamping experiment showing baseline (0–60 s) and motion (100–160 s) segments for one participant, boxed to match zoomed–in views (**b**) and (**d**). **b.** 1-minute baseline segment used for automatic subspace reconstruction (ASR) calibration, where the participant was asked to stand motionless with eyes closed. Minor artifacts are seen and retained for ASR calibration. **c.** PSD for the baseline segment, showing mean channel power and standard deviation. 40 Hz ASSR and 31.2 Hz impedance measurement peaks are seen in the passband. **d.** 1-minute motion segment showing artifacts corresponding to the participant's stamping action, which is input to the ASR filter. **e.** The same 1-minute motion segment processed with ASR. Artifact amplitudes are seen to be reduced. **f.** PSDs comparing mean channel power for the motion segment before and after running ASR. The noise floor is reduced by ASR, making the 40 Hz ASSR and 31.2 Hz impedance measurement peaks visible again.

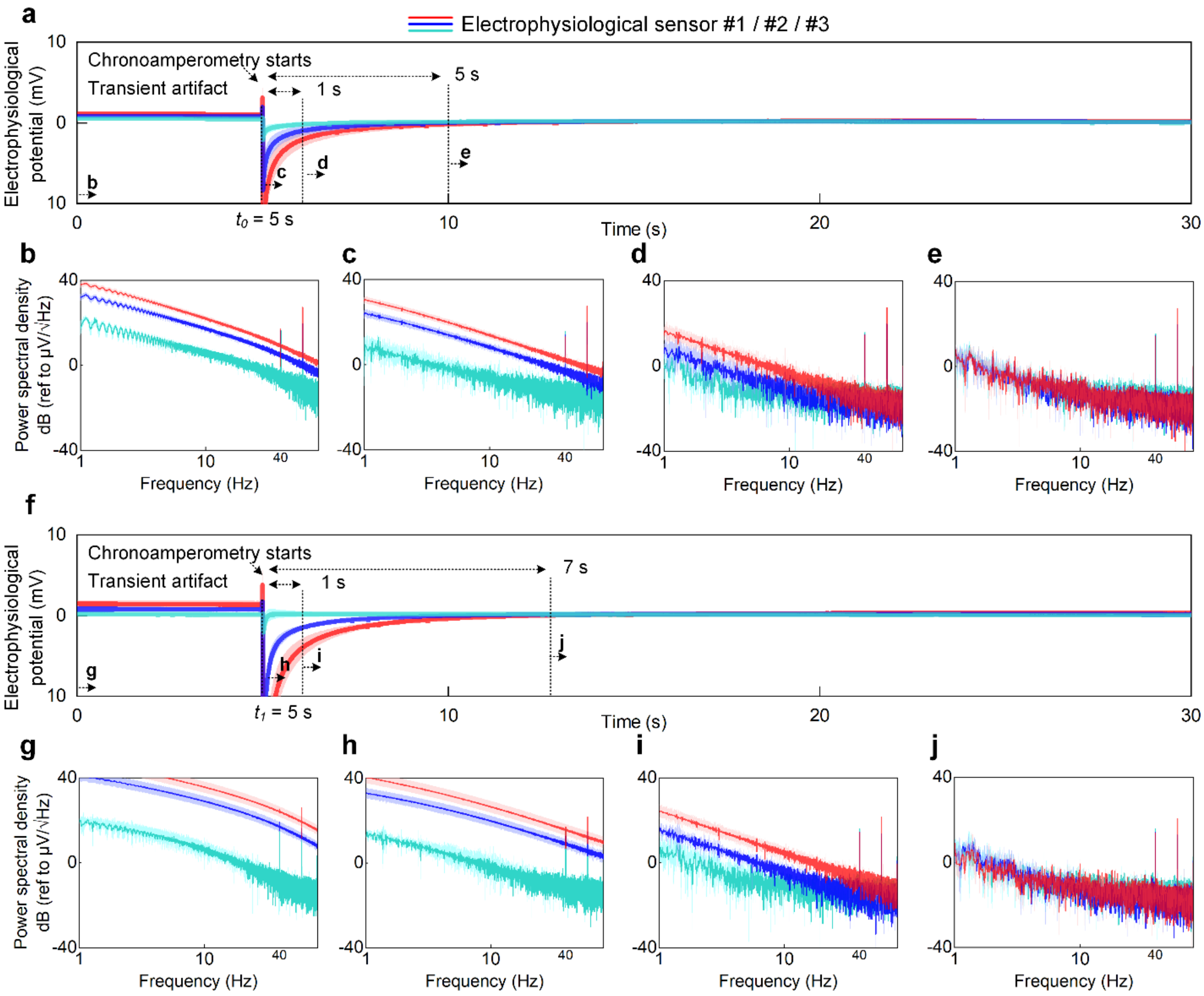

Supplementary Fig. 9 I Crosstalk characterization for lactate recording's effect on EEG recording. Here EEG 40 Hz ASSR power spectrum density analyses (without time-domain averaging, log-log scale) are conducted with the lactate CA crosstalk in a less sweaty condition (**a**–**e**) and a sweaty condition (**f**–**j**). (**a,f**) show 0–30 s zoom-in view of **Extended Data Fig. 9e** and **Extended Data Fig. 9h**, demonstrating a transient artifact along with a discharging phase introduced to the EEG data stream. (**b**–**e**) and (**g**–**j**) show averaged PSDs of the three 40 Hz ASSR measurements obtained by using different data rejection periods. Here t_0 = 5 s, which is the timing of the start of the CA recording and the transient artifact. t_e = 70 s, which is the timing of the end of the EEG recording. Different portions of the EEG times series were used under different artifact blanking conditions (t_s and t_e are the starting and ending timings): (**b,g**) From t_s = 0 s (Start of the EEG recording) to t_e. (**c,h**) From t_s = t_0 +15 ms (After the apex of the CA transient artifact) to t_e. (**d,i**). From t_s = t_1 +1 s to t_e. (**e**) From t_s = t_1 +5 s or (**j**) t_s = t_1 +7 s (The three electrophysiological sensors' time series starts to plateau) to t_e. The participant-averaged mean and standard deviation of the measured electrophysiological time series and PSD, are represented by solid lines and shaded bands, respectively.

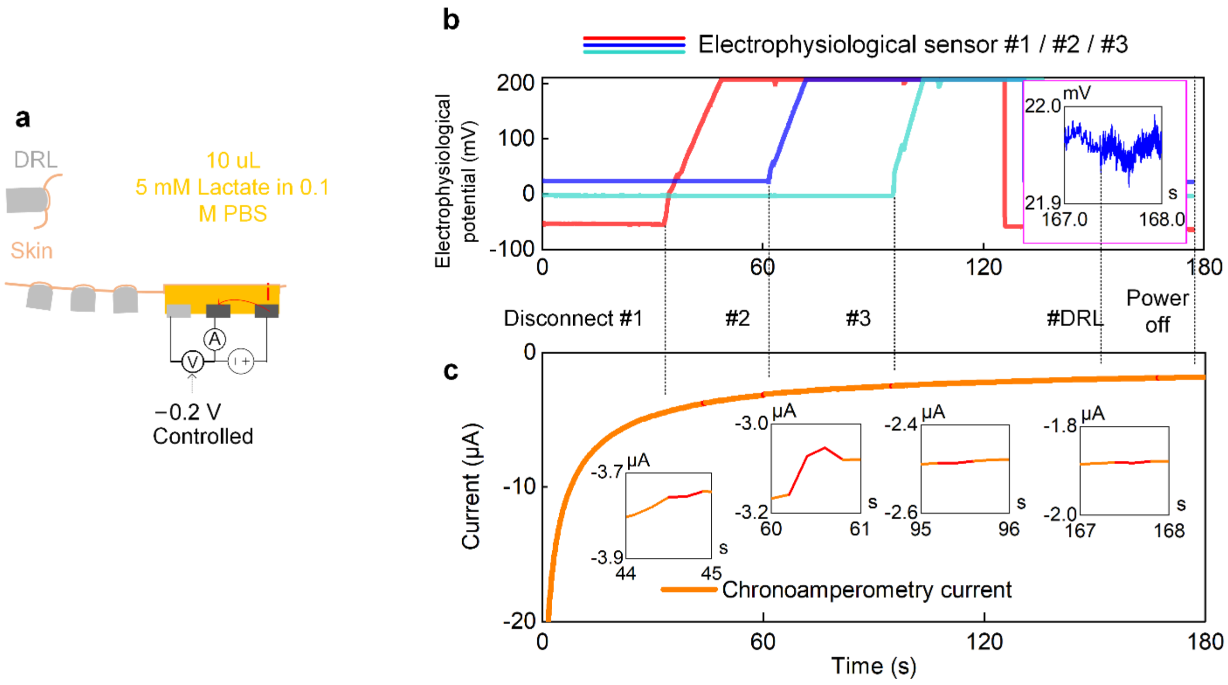

Supplementary Fig. 10 | Crosstalk characterization for EEG recording's effect on Lactate recording. **a.** Setup to explore how EEG measurement affects lactate measurement. **b.** EEG measurement of a 180-s session, during which the following actions were performed: disconnecting electrophysiological sensor #1, disconnecting electrophysiological sensor #2, disconnecting electrophysiological sensor #3, reconnecting all the electrophysiological sensors and disconnecting DRL, power off the EEG DAQ instrument. **c.** The synchronous 180-s lactate sensing CA measurement.

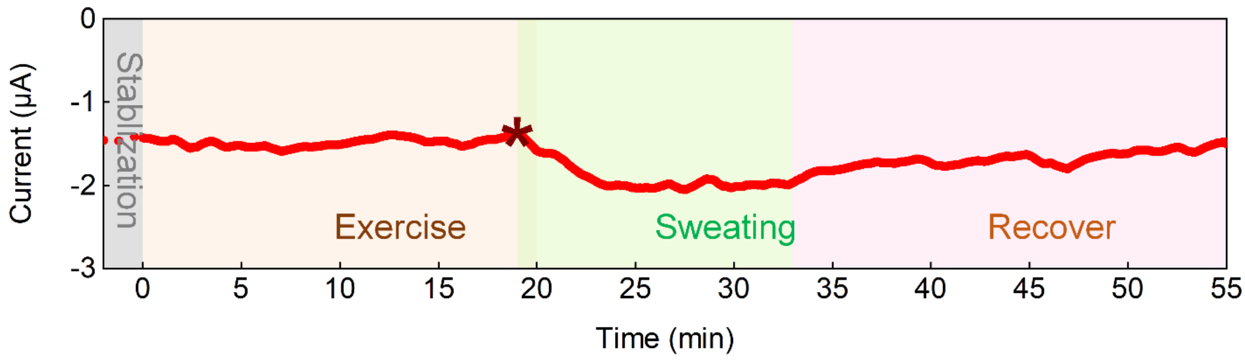

Supplementary Fig. 11 | Extended analysis of the lactate CA current during the exercise. The extended 1-hour recording of the lactate CA current from participant 5, who started to sweat close to the end of the exercise and experienced a later lactate CA current recovery phase. The 30-min sub dataset of the recording has been reported in **Fig. 4e** and **Extended Data Fig. 10o** as participant 5.

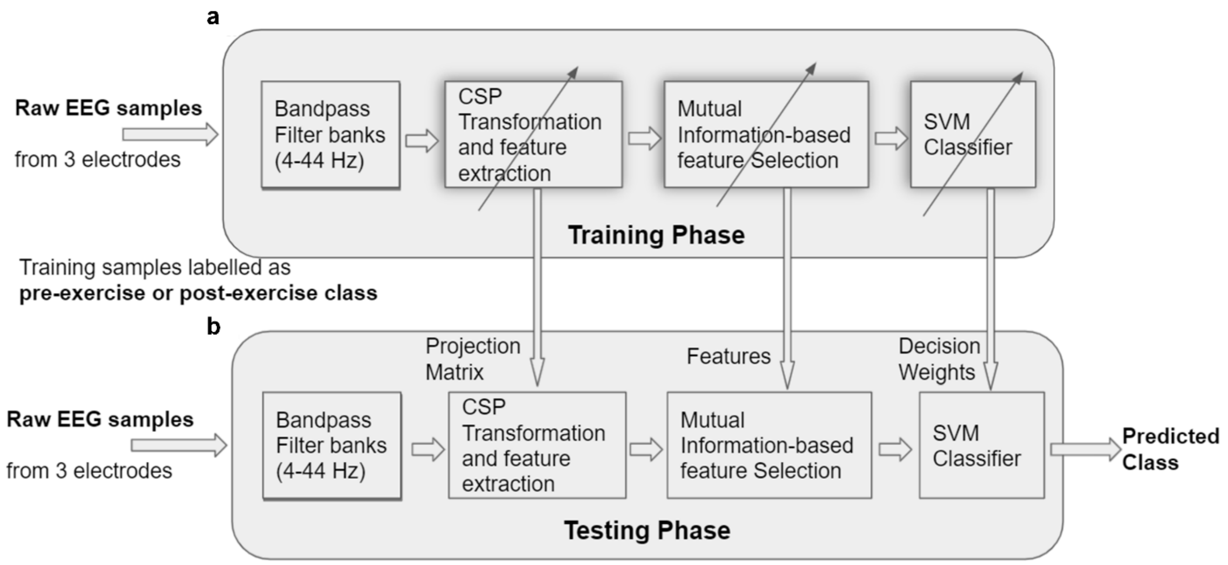

Supplementary Fig. 12 | Filter-bank-based common-spatial-pattern method data processing pipeline. **a**. Training phase for FBCSP. In this Phase, the CSP projection matrix, discriminative features selection, and support-vector-machine (SVM) classifier weights are obtained. Two sets of signals were provided to the pipeline with classes labeled as either (i) Pre-exercise and Post-exercise-immediate or (ii) Pre-exercise and Post-exercise-relaxed. **b.** Testing Phase. In this phase, CSP projection matrix, feature selection, and SVM parameters are obtained. During the testing phase, we evaluated unseen EEG samples and predicted the class.

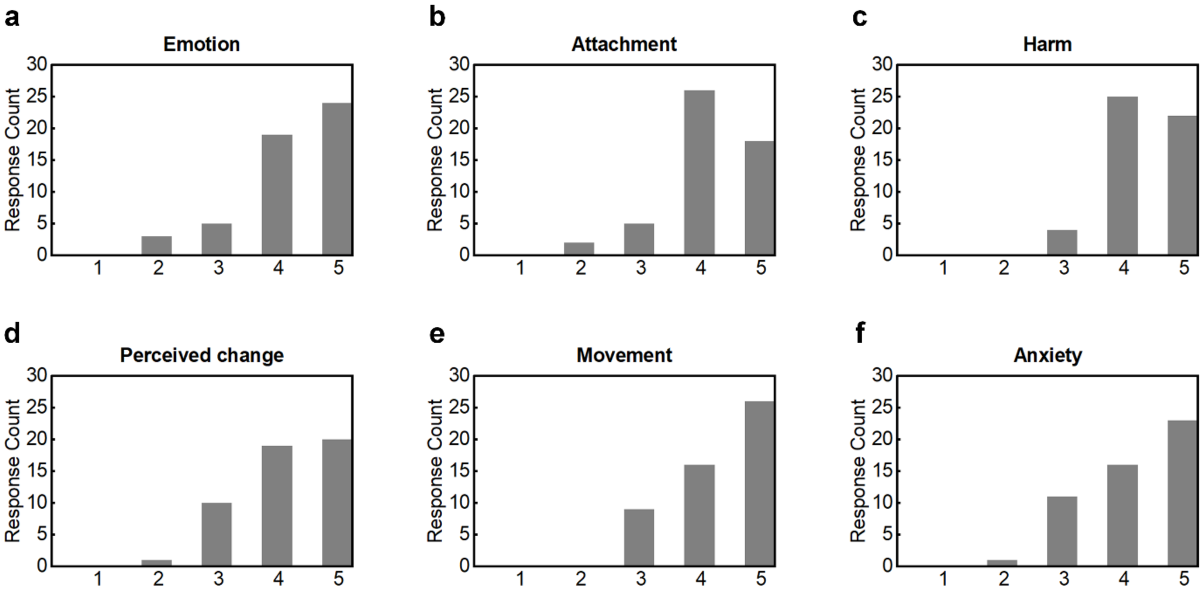

Supplementary Fig. 13 | Results of the usability and wearability questionnaires. Response count of **a.** "Emotion" question, **b.** "Attachment" question, **C.** "Harm" question, **d.** "Perceived change" question, **e.** "Movement" question, and **f.** "Anxiety" question. In total, 51 responses were received.

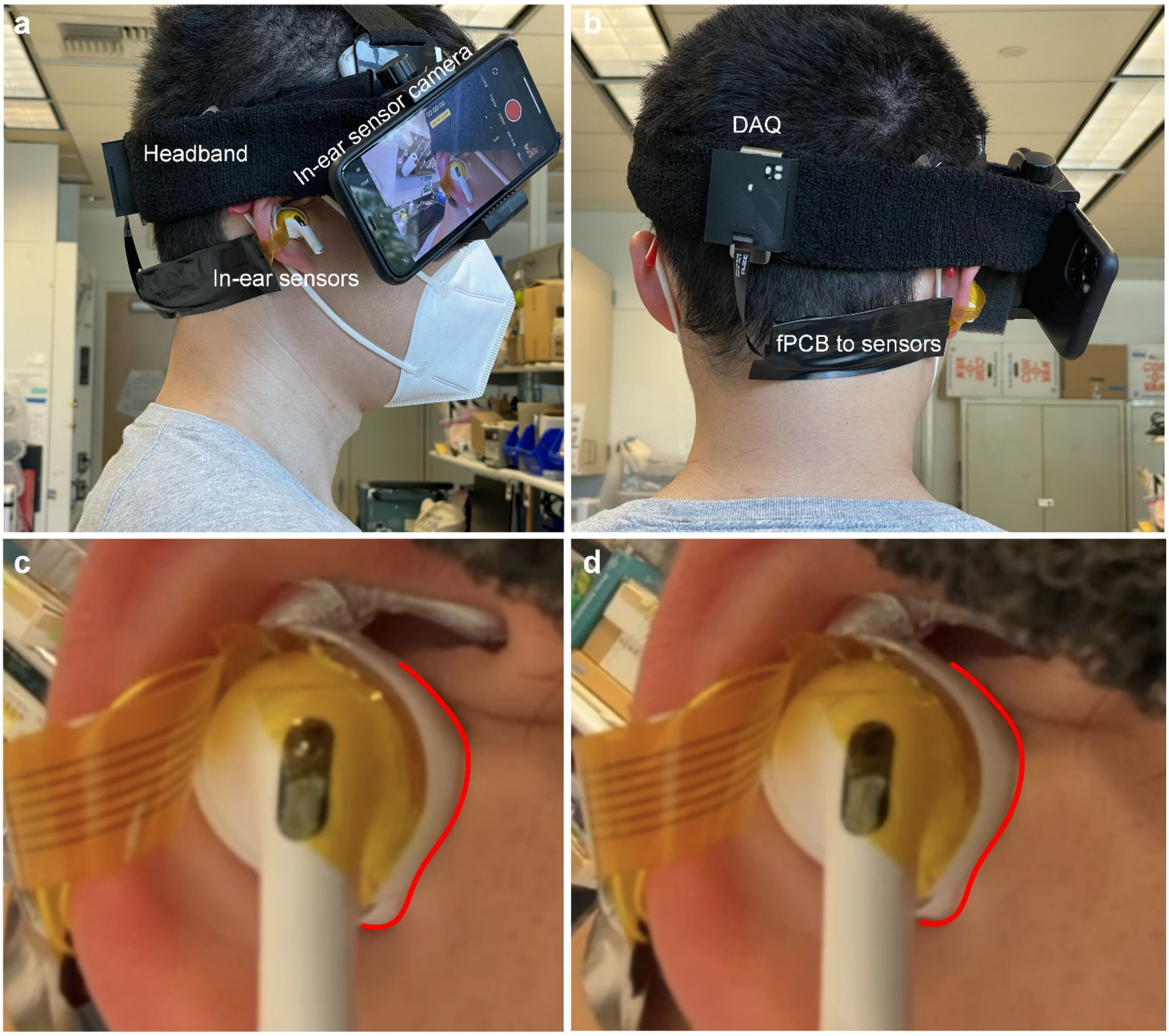

Supplementary Fig. 14 | Validation of mechanical stability of in-ear sensors in Supplementary Video 3. a. Side view showing the over-the-head camera for a close-up head-referenced view of the in-ear sensors and the earphone. **b.** Rear view showing the DAQ and its connection to the in-ear sensors via flexible PCB. The in-ear sensors' stability was evaluated by comparing the **c.** first and **d.** last frame of Supplementary Video 3 showing the earphone and cable extensions of the sensors at the same view angle with no discernable change in the contours of their contact to the ear.

Supplementary Tables

Supplementary Table 1 | Comparison between this work and other representative in-ear sensing systems.

	This work	***Kappel et al, 2019*** [24]	***Kaveh et al, 2020*** [25]	***Sintotskiy et al, 2020*** [65]	***Goverdovsky et al, 2017*** [29]	***Da He et al, 2015*** [30]	***Matsumoto et al, 2019*** [42]	***Bruno et al, 2019*** [66]
Functionality	EEG + Lactate	EEG	EEG	EEG	EEG + ECG (Electrocardiography) + respiration	ECG + BCG + PPG	Temperature / Sweat rate Na^+ concentration	ECG + Lactate + PH + ACC Sweat
Earpiece style	Generic	Custom	Generic	Custom	Generic	Custom	Generic	Generic (not in-ear)
Earphone or Mic	Integrated with commercial earphones	Sound tube for external mic	External mic	None	Integrated Mic	None	Separate	None
Number of channels	6 (3 electrophysiological and 3 electrochemical)+DRL+REF	5 EEG+REF	4 EEG+REF+DRL	1 EEG	2 EEG	2 ECG	-	1 ECG + 3 electrochemical
Sensor type	Fabricated and assembled	Fabricated and assembled	Fabricated and assembled	Fabricated and assembled	Fabricated and assembled	Commercial components	Commercial components	Commercial components
Electrode	Stretchable Ag Stretchable PB	IrO_2	Ag coating	Ag/AgCl	Conductive cloth	-	-	Stretchy silver-plated nylon
Electrode-ear impedance @ 50 Hz and size	386.45 kΩ 3D electrodes Planar size: 13 mm^2	1.1 MΩ	377 kΩ 60 mm^2	-	Did not specify size and frequency	-	-	< MΩ range,
Alpha modulation ratio (V^2/V^2)	2.44	1.2 6.6 maximum	2.17	-	Recorded but not reported	-	-	-
ASSR SNR (dB)	8.98 19.69maximum	Approximately 15	5.94 6.80 maximum	-	Approximately 10–15	-	-	-
Wireless	Yes	No	Yes	No	No	Yes	No	Yes

Supplementary Table 2 I Accuracy of FBCSP-based brain state classification of each 5 participants' pre- and post-exercise in-ear EEG data. For every participant, both the post-exercise-immediate state and post-exercise-relaxed state are classified with respect to the pre-exercise state as the baseline. We perform this analysis under both eyes open and eyes closed conditions. We also apply FBCSP using a variable number of frequency bands with diff low-pass cutoff and high-pass cutoff frequencies, with a goal to detect the bands with the number of features useful for the classification task. For most participants, using more frequency bands ended up having a higher classification accuracy. The classification results appear to be reproducible as all participants have similar accuracy across different frequency bands and eyes open/closed state.

Frequency bands	Participants	Accuracy<br>Eyes open<br>Fully Relaxed	 <br>Immediately Post-Exercise	 Eyes closed<br>Fully Relaxed	 <br>Immediately Post-Exercise
4-28 Hz (3 bands)	Participant 1	63.71%	92.00%	65.43%	96.29%
	Participant 2	66.00%	90.85%	65.42%	94.85%
	Participant 3	60%	74.85%	64.28%	77.14%
	Participant 4	62.57%	84.85%	62.57%	91.14%
	Participant 5	68.00%	79.43%	58.57%	82.85%
4-36 Hz (4 bands)	Participant 1	67.14%	92.00%	65.43%	96.29%
	Participant 2	64.28%	92.28%	71.71%	94.28%
	Participant 3	60%	80.85%	64.28%	85.14%
	Participant 4	57.42%	87.71%	57.42%	89.71%
	Participant 5	68.00%	79.43%	61.71%	86.28%
4-44 Hz (5 bands)	Participant 1	65.42%	93.14%	72.57%	96.86%
	Participant 2	67%	93.71%	70.86%	97.14%
	Participant 3	60%	85.14%	64.86%	85.14%
	Participant 4	60.57%	87.71%	60.57%	89.71%
	Participant 5	66.28%	89.14%	61.71%	88.57%
12-44Hz (4 bands)	Participant 1	65.42%	93.14%	72.57%	96.86%
	Participant 2	65%	93.71%	70.86%	97.14%
	Participant 3	59.71%	85.14%	60.86%	85.14%
	Participant 4	60.57%	87.71%	60.57%	86.57%
	Participant 5	63.42%	89.14%	61.71%	88.85%
20-44 Hz (3 bands)	Participant 1	68.00%	91.71%	72.57%	93.14%
	Participant 2	65%	93.71%	70.86%	97.14%
	Participant 3	59.71%	85.14%	60.86%	85.14%
	Participant 4	60.57%	88.85%	60.57%	86.57%
	Participant 5	63.42%	89.14%	65.43%	88.57%

Supplementary Table 3 | Statistics of human participants who participated in this study.

	Age	Gender	Health condition	Recruitment
Participant 1	31	Male	Healthy	Self-volunteered
Participant 2	29	Male	Healthy	Self-volunteered
Participant 3	24	Male	Healthy	Online platform
Participant 4	30	Female	Healthy	Online platform
Participant 5	24	Male	Healthy	Online platform
Participant 6	28	Male	Healthy	Self-volunteered
Participant 7	32	Female	Healthy	Online platform
Participant 8	23	Male	Healthy	Online platform
Participant 9	22	Male	Healthy	Self-volunteered
Participant 10	30	Male	Healthy	Self-volunteered

Supplementary Table 4 | Details of human participant participation for each experiment

Experimental section	Sub-experiment	Participant #	Condition
In-ear sweat mapping		Participant 6	Right ear
		Participant 7	Left ear
		Participant 8	Right ear
Electrophysiological measurements	Ear-electrode impedance	Participant 1	Right ear
		Participant 2	Left ear
	Electrode DC offset	Participant 1	Two ears
		Participant 2	Two ears
	Alpha modulation	Participant 1	Right ear
		Participant 2	Right ear
		Participant 3	Left ear
		Participant 4	Left ear
	ASSR	Participant 1	Right ear
		Participant 2	Right ear
		Participant 3	Left ear
		Participant 5	Left ear
		Participant 4	Two ears
	Eye movement	Participant 1	Two ears
		Participant 2	Two ears
	Simultaneous electrophysiological	Participant 1	Right ear
	Motion artifact	Participant 1	Right ear
Electrophysiological measurements validation with EEG headset	Alpha modulation	Participant 1	Two ears
		Participant 2	Two ears
	ASSR	Participant 1	Two ears
		Participant 2	Two ears
	Eye movement	Participant 1	Two ears
		Participant 2	Two ears
Electrochemical measurements and validation with blood lactate meter		Participant 6	One ear
		Participant 9	One ear
		Participant 10	One ear
Co-sensing crosstalk characterization		Participant 1	Two ears
		Participant 6	Left ear
Co-sensing experiment		Participant 1	Right ear
		Participant 6	Left ear
		Participant 8	Right ear
		Participant 9	Right ear
		Participant 10	Left ear

Supplementary Videos

Supplementary Video 1 I Eye movement sensing demonstration
During the video, the participant performed 4 eyeball movements: glancing up, glancing down, glancing left, and glancing right, then a heavy blinking and a normal blinking. The participant wore two integrated in-ear sensors in both ears, with one channel of ipsilateral reference measurement and one channel of contralateral referencing measurement shown in the video. During recording, the waveform was shown in the BioCapture software in real-time. For demonstration, the waveforms removed the DC offset and 60 Hz powerline noise in real-time by applying a 4 order Butterworth bandpass filter (0.5 Hz to 20 Hz) to both channels. The waveform in the video differed from **Extended Data Fig. 5a**–**c** due to the different filters applied. The waveform shown in **Extended Data Fig. 5a**–**c** removed DC offset by mean subtraction and applied a 60 Hz notch filter to illustrate the measured eye movement signals with high fidelity.
The video demonstrated the finding in the eye movement results section: ipsilateral referencing has better resilience to eyeball movement artifacts than contralateral referencing measurements. Both sensing modalities measured blink signals, but ipsilateral referencing still recorded a smaller amplitude.

Supplementary Video 2 I Lactate sensing demonstration
During the video, the participant performed a 20-min cycling with the in-ear integrated sensors in one ear. The lactate CA current was shown live in the PSTrace software and demonstrated the sweating process live during the recording. The video demonstrated real-time, raw and unprocessed lactate CA current, and a ~0.5 μA increase of CA current with the secretion of exercise-induced sweat lactate.

Supplementary Video 3 I Mechanical stability demonstration
Supplementary Fig. 15 shows the setup and summarizes results of the mechanical stability characterization experiment shown in Supplementary Video 3. The participant wore in-ear integrated sensors and earphones on both ears, and a custom designed DAQ system was attached to the sensors to show how the entire sensing system could be effectively miniaturized[67]. To obtain a close-up view of the sensors and to directly observe any relative displacements between the in-ear sensors and the ear, an additional over-the-head camera was mounted to the participant providing a steady frame of reference fixated to the head. The individual engaged in a number of intense physical activities throughout the video, including running and jumping. Neither the sensors nor the earphones detached during the physical activities, and the in-ear integrated sensors remained mechanically stable throughout the entire session, as demonstrated by close matching observed in the in-ear sensor-ear contact contours across the first and last frames of the video.

Supplementary references

17 Chi, Y. M., Jung, T.-P. & Cauwenberghs, G. Dry-contact and noncontact biopotential electrodes: Methodological review. *IEEE Reviews in Biomedical Engineering* **3**, 106-119 (2010).

24 Kappel, S. L., Rank, M. L., Toft, H. O., Andersen, M. & Kidmose, P. Dry-Contact Electrode Ear-EEG. *IEEE Transactions on Biomedical Engineering* **66**, 150-158 (2019). https://doi.org:10.1109/tbme.2018.2835778

25 Kaveh, R. *et al.* Wireless User-Generic Ear EEG. *IEEE Transactions on Biomedical Circuits and Systems* **14**, 727-737 (2020).

29 Goverdovsky, V. *et al.* Hearables: Multimodal physiological in-ear sensing. *Scientific Reports* **7**, 1-10 (2017).

30 Da He, D., Winokur, E. S. & Sodini, C. G. An ear-worn vital signs monitor. *IEEE Transactions on Biomedical Engineering* **62**, 2547-2552 (2015).

40 Levander, M. S. & Grodzinsky, E. Variation in normal ear temperature. *The American journal of the medical sciences* **354**, 370-378 (2017).

41 Gray, R., Sharma, A. & Vowler, S. Relative humidity of the external auditory canal in normal and abnormal ears, and its pathogenic effect. *Clinical otolaryngology* **30**, 105-111 (2005).

42 Matsumoto, K. *et al.* in *2019 41st Annual International Conference of the IEEE Engineering in Medicine and Biology Society (EMBC).* 7049-7055 (IEEE).

51 Akhtar, S. S. An Integrated Approach to Design and Develop High-Performance Polymer-Composite Thermal Interface Material. *Polymers* **13**, 807 (2021).

52 Matmatch. *Urethane thermoplastic elastomer (TPU)*, <https://matmatch.com/materials/mbas116-urethane-thermoplastic-elastomer-tpu> (2021).

53 Greer, J. R. & Street, R. A. Mechanical characterization of solution-derived nanoparticle silver ink thin films. *Journal of Applied Physics* **101**, 103529 (2007).

54 AmericanElements. *Silver Ink*, <https://www.americanelements.com/silver-ink-7440-22-4> (2021).

55 Sempionatto, J. R., Moon, J.-M. & Wang, J. Touch-Based Fingertip Blood-Free Reliable Glucose Monitoring: Personalized Data Processing for Predicting Blood Glucose Concentrations. *ACS Sensors* **6**, 1875-1883 (2021).

56 Strijkstra, A. M., Beersma, D. G., Drayer, B., Halbesma, N. & Daan, S. Subjective sleepiness correlates negatively with global alpha (8–12 Hz) and positively with central frontal theta (4–8 Hz) frequencies in the human resting awake electroencephalogram. *Neuroscience letters* **340**, 17-20 (2003).

57 Röddiger, T. *et al.* Sensing with Earables: A Systematic Literature Review and Taxonomy of Phenomena. *Proceedings of the ACM on Interactive, Mobile, Wearable and Ubiquitous Technologies* **6**, 1-57 (2022).

58 Seok, D., Lee, S., Kim, M., Cho, J. & Kim, C. Motion artifact removal techniques for wearable EEG and PPG sensor systems. *Frontiers in Electronics* **2**, 685513 (2021).

59 Gorjan, D., Gramann, K., De Pauw, K. & Marusic, U. Removal of movement-induced EEG artifacts: current state of the art and guidelines. *Journal of neural engineering* (2022).

60 Kotte, S. & Dabbakuti, J. R. K. K. Methods for removal of artifacts from EEG signal:A review. *Journal of Physics: Conference Series* **1706** (2020). https://doi.org:10.1088/1742-6596/1706

61 Hsu, S.-H., Mullen, T. R., Jung, T.-P. & Cauwenberghs, G. Real-Time Adaptive EEG Source Separation Using Online Recursive Independent Component Analysis. *IEEE Transactions on Neural Systems and Rehabilitation Engineering* **24**, 309-319 (2016). https://doi.org:10.1109/tnsre.2015.2508759

62 Mullen, T. R. *et al.* Real-time neuroimaging and cognitive monitoring using wearable dry EEG. *IEEE Transactions on Biomedical Engineering* **62**, 2553-2567 (2015).https://doi.org:10.1109/tbme.2015.2481482

63 Keogh, A., Argent, R., Anderson, A., Caulfield, B. & Johnston, W. Assessing the usability of wearable devices to measure gait and physical activity in chronic conditions: a systematic review. *Journal of NeuroEngineering and Rehabilitation* **18**, 1-17 (2021).

64 Knight, J. F. & Baber, C. A tool to assess the comfort of wearable computers. *Human factors* **47**, 77-91 (2005).

65 Sintotskiy, G. & Hinrichs, H. In-ear-EEG–a portable platform for home monitoring. *Journal of Medical Engineering & Technology* **44**, 26-37 (2020).

66 Gil, B., Anastasova, S. & Yang, G. Z. A smart wireless Ear-Worn device for cardiovascular and sweat parameter monitoring during physical exercise: design and performance results. *Sensors* **19**, 1616 (2019).

67 Paul, A., Lee, M. S., Xu, Y., Deiss, S. R. & Cauwenberghs, G. A Versatile In-Ear Biosensing System

and Body-Area Network for Unobtrusive Continuous Health Monitoring. *IEEE Transactions on Biomedical Circuits and Systems*, 1-12 (2023). https://doi.org:10.1109/tbcas.2023.3272649.
