# Supplementary material for: In-ear integrated sensor array for the continuous monitoring of brain activity and of lactate in sweat
Source: Nat Biomed Eng. 2023 Sep 28;7(10):1307–20. doi: 10.1038/s41551-023-01095-1 (PMC10589098; doi:10.1038/s41551-023-01095-1)
Supplement: Supplementary file 2 — Reporting Summary [file 41551_2023_1095_MOESM2_ESM.pdf]

nature portfolio

Corresponding author(s): Gert Cauwenberghs, Joseph Wang, Sheng Xu, Patrick Mercier

Last updated by author(s): Jun 20, 2023

Reporting Summary

Nature Portfolio wishes to improve the reproducibility of the work that we publish. This form provides structure for consistency and transparency in reporting. For further information on Nature Portfolio policies, see our Editorial Policies and the Editorial Policy Checklist.

Statistics

For all statistical analyses, confirm that the following items are present in the figure legend, table legend, main text, or Methods section.

n/a	Confirmed	
☐	☒	The exact sample size (n) for each experimental group/condition, given as a discrete number and unit of measurement
☐	☒	A statement on whether measurements were taken from distinct samples or whether the same sample was measured repeatedly
☒	☐	The statistical test(s) used AND whether they are one- or two-sided<br>*Only common tests should be described solely by name; describe more complex techniques in the Methods section.*
☒	☐	A description of all covariates tested
☐	☒	A description of any assumptions or corrections, such as tests of normality and adjustment for multiple comparisons
☐	☒	A full description of the statistical parameters including central tendency (e.g. means) or other basic estimates (e.g. regression coefficient) AND variation (e.g. standard deviation) or associated estimates of uncertainty (e.g. confidence intervals)
☒	☐	For null hypothesis testing, the test statistic (e.g. F, t, r) with confidence intervals, effect sizes, degrees of freedom and P value noted<br>*Give P values as exact values whenever suitable.*
☒	☐	For Bayesian analysis, information on the choice of priors and Markov chain Monte Carlo settings
☒	☐	For hierarchical and complex designs, identification of the appropriate level for tests and full reporting of outcomes
☐	☒	Estimates of effect sizes (e.g. Cohen's d, Pearson's r), indicating how they were calculated

Our web collection on statistics for biologists contains articles on many of the points above.

Software and code

Policy information about availability of computer code

Data collection	Electrophysiological data (EEG, EOG, Electrode DC offset) were collected from the sensors using the BioCapture software (version 5.5.640) (Great Lakes Neurotechnologies). In the validation test, electrophysiological data (EEG, EOG) collected from the Cognionics headset used the CGX 2021 (Cognionics) software.<br><br>Electrode-ear impedance and chronoamperometry (including lactate measurement) data were collected from the sensors using the PSTrace 5.9 (Palmsense). In the validation test, lactate data collected from the NOVA Biomedical blood-lactate meter used the CHI instruments.
Data analysis	Electrophysiological data (EEG, EOG, Electrode DC offset), electrode-ear impedance data and chronoamperometry data (including lactate measurement) were analysed and visualized using Matlab R2021b (Mathworks) and OriginPro 2021 (Originlab). ASR EEG artifact algorithm was implemented using Matlab R2021b.<br>Filter-bank-based common-spatial-pattern method for EEG cognitive-state classification used scripts coded in Python.<br><br>Custom codes for electrophysiological signal analysis, for the automatic subspace reconstruction (ASR) algorithm, and for the filter-bank-based common-spatial-pattern (FBCSP) method, are available at https://doi.org/10.1038/zenodo.8193117.

For manuscripts utilizing custom algorithms or software that are central to the research but not yet described in published literature, software must be made available to editors and reviewers. We strongly encourage code deposition in a community repository (e.g. GitHub). See the Nature Portfolio guidelines for submitting code & software for further information.

Data

Policy information about availability of data

All manuscripts must include a data availability statement. This statement should provide the following information, where applicable:

- Accession codes, unique identifiers, or web links for publicly available datasets
- A description of any restrictions on data availability
- For clinical datasets or third party data, please ensure that the statement adheres to our policy

All data supporting the results in this study are available within the paper and its Supplementary Information. Source data for the figures are provided with this paper, and are available in figshare at https://doi.org/10.6084/m9.figshare.22829051.

Human research participants

Policy information about studies involving human research participants and Sex and Gender in Research.

Reporting on sex and gender	The gender of all participants is indicated in Supplementary Table 3.
Population characteristics	In-ear sweat gland mapping: 3 participants and 3 recording sessions.<br>Ear-electrode impedance experiment: 2 participants and 6 measurement sessions for in total 6 electrophysiological electrodes.<br>Electrode DC offset and EOG: 2 participants and 12 measurement sessions for in total 12 electrophysiological electrodes (two ears per participant).<br>Alpha modulation and auditory steady state response: 4 participants and 12 measurement sessions for in total 12 electrophysiological electrodes.<br>Simultaneous multi-modal electrophysiological recording: 1 participant and 1 recording session for in total 3 electrophysiological electrodes (EEG + EOG, EEG + EDA).<br>Electrophysiological validation with the dry EEG headset: 2 participants and 4 recording sessions for in total 12 electrophysiological electrodes (Alpha, ASSR, EOG).<br>Motion artifact analysis: 1 participant and 1 recording session for in total 3 electrophysiological electrodes.<br>On-body lactate sensing assessment: 3 participants and 3 recording sessions.<br>Crosstalk analysis of electrophysiological and electrochemical sensing: 2 participants and 3 recording sessions (one participant with both ears).<br>EEG and lactate co-sensing experiment: 5 participants and 5 recording sessions.
Recruitment	Recruitment information was shared via flyers and an online platform. No age, race/ethnicity or gender-based participant-exclusion criteria were used. The participants recruited were healthy, and were asked to fill out an online qualification form before participating in the experiment.
Ethics oversight	University of California San Diego Institutional Review Boards.

Note that full information on the approval of the study protocol must also be provided in the manuscript.

Field-specific reporting

Please select the one below that is the best fit for your research. If you are not sure, read the appropriate sections before making your selection.

☒ Life sciences ☐ Behavioural & social sciences ☐ Ecological, evolutionary & environmental sciences

For a reference copy of the document with all sections, see nature.com/documents/nr-reporting-summary-flat.pdf

Life sciences study design

All studies must disclose on these points even when the disclosure is negative.

Sample size	Please see 'population characteristics' above.
Data exclusions	To avoid co-sensing crosstalk, we didn't include EEG data immediately after the onset of lactate chronoamperometry measurements. Detailed justification of the exclusions can be found in the Supplementary Information.
Replication	The study validated the integration of electrophysiological and electrochemical sensing in the ear. The result was dependent on the body condition for each experiment session and was thus not replicated. However, for all the electrophysiological and electrochemical validation experiments and combined EEG and lactate-sensing experiments, the measurements were taken from multiple participants, to demonstrate repeatibility.
Randomization	The human participants for each experiment were selected randomly.

Blinding	No blinding experiments were carried out. The data were directly processed after collection.

Reporting for specific materials, systems and methods

We require information from authors about some types of materials, experimental systems and methods used in many studies. Here, indicate whether each material, system or method listed is relevant to your study. If you are not sure if a list item applies to your research, read the appropriate section before selecting a response.

Materials & experimental systems

n/a	Involved in the study
☒	☐ Antibodies
☒	☐ Eukaryotic cell lines
☒	☐ Palaeontology and archaeology
☒	☐ Animals and other organisms
☒	☐ Clinical data
☒	☐ Dual use research of concern

Methods

n/a	Involved in the study
☒	☐ ChIP-seq
☒	☐ Flow cytometry
☒	☐ MRI-based neuroimaging
